# LKB1 controls inflammatory potential through CRTC2-dependent histone acetylation

**DOI:** 10.1016/j.molcel.2023.04.017

**Published:** 2023-05-11

**Authors:** Shelby E. Compton, Susan M. Kitchen-Goosen, Lisa M. DeCamp, Kin H. Lau, Batsirai Mabvakure, Matthew Vos, Kelsey S. Williams, Kwok-Kin Wong, Xiaobing Shi, Scott B. Rothbart, Connie M. Krawczyk, Russell G. Jones

**Affiliations:** 1 Department of Metabolism and Nutritional Programming, Van Andel Institute, Grand Rapids, MI, USA; 2 Metabolism and Nutrition (MeNu) Program, Van Andel Institute, Grand Rapids, MI, USA; 3 Bioinformatics and Biostatistics Core, Van Andel Institute, Grand Rapids, MI, USA; 4 Laura and Isaac Perlmutter Cancer Center, NYU Langone Health, New York, NY, USA; 5 Department of Epigenetics, Van Andel Institute, Grand Rapids, MI, USA; 6 Lead contact

## Abstract

Deregulated inflammation is a critical feature driving the progression of tumors harboring mutations in the liver kinase B1 (LKB1), yet the mechanisms linking LKB1 mutations to deregulated inflammation remain undefined. Here, we identify deregulated signaling by CREB-regulated transcription coactivator 2 (CRTC2) as an epigenetic driver of inflammatory potential downstream of LKB1 loss. We demonstrate that LKB1 mutations sensitize both transformed and non-transformed cells to diverse inflammatory stimuli, promoting heightened cytokine and chemokine production. LKB1 loss triggers elevated CRTC2-CREB signaling downstream of the salt-inducible kinases (SIKs), increasing inflammatory gene expression in LKB1-deficient cells. Mechanistically, CRTC2 cooperates with the histone acetyltransferases CBP/p300 to deposit histone acetylation marks associated with active transcription (i.e., H3K27ac) at inflammatory gene loci, promoting cytokine expression. Together, our data reveal a previously undefined anti-inflammatory program, regulated by LKB1 and reinforced through CRTC2-dependent histone modification signaling, that links metabolic and epigenetic states to cell-intrinsic inflammatory potential.

## In brief

Deregulated inflammation is a critical feature driving the progression of tumors harboring mutations in liver kinase B1 (LKB1). Compton et al. identify a mechanism by which LKB1 epigenetically regulates cellular inflammatory potential through CRTC2-dependent histone acetylation at inflammatory gene loci.

## Graphical abstract

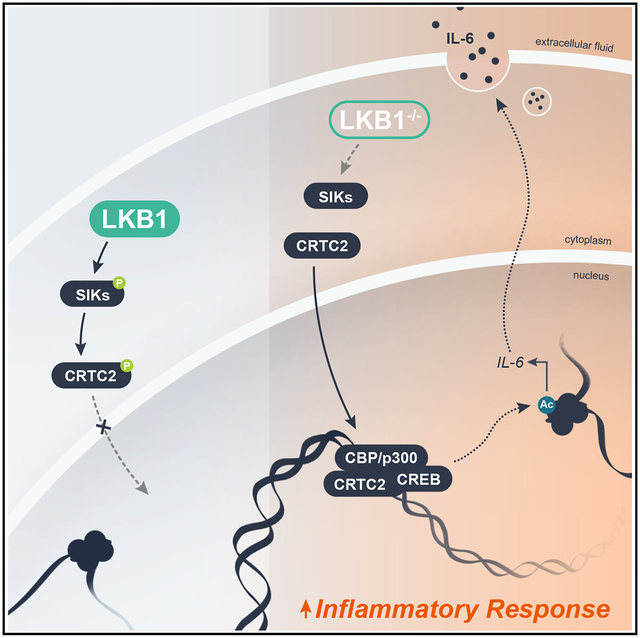

## INTRODUCTION

Liver kinase B1 (LKB1) is a serine/threonine kinase with key regulatory roles in metabolism, cell polarity, cell growth, and proliferation.^1^ Loss of LKB1 function (via mutations in the serine/threonine kinase 11 [*STK11*] gene) is a common occurrence in human cancer,^2–6^ while heterozygous germline mutations in *STK11* predispose humans and mice to the development of Peutz-Jeghers syndrome (PJS),^7–10^ an autosomal dominant disease characterized by the development of hamartomatous polyps in the gastrointestinal (GI) tract.^11^ In addition to GI issues, PJS patients carry a 93% cumulative risk of developing GI, breast, pancreatic, and gynecological cancers by age 65—a cancer risk similar to that in *BRCA1/2* carriers.^12^ Loss of LKB1 function is among the most common genetic mutations in human epithelial cancers, including non-small cell lung cancer (NSCLC), where it is frequently co-mutated with *KRAS*,^13–15^ pancreatic cancer,^6,16^ and cervical cancer.^4,17^ LKB1 mutations are associated with poor patient outcomes and resistance to both conventional chemotherapy and immune checkpoint inhibitors (ICIs).^13,18–20^ Despite these observations, how LKB1 mutations lead to the development of PJS polyps and malignant tumors remains poorly understood, limiting treatment options and increasing patient mortality.

LKB1 regulates the activity of a series of downstream kinases belonging to the AMP-activated protein kinase (AMPK) and AMPK-related kinase (ARK) families. Many of the tumor suppressor functions of LKB1 have been attributed to its negative regulation of the mechanistic target of rapamycin (mTOR) complex 1 (mTORC1) signaling pathway, mediated in part by LKB1-dependent phosphorylation and activation of AMPK.^21–23^ However, despite elevated mTORC1 activity in LKB1 mutant tumors,^21,24^ the mTORC1 inhibitor rapamycin has shown modest effects on the growth of pre-existing GI polyps in pre-clinical PJS mouse models.^25–27^ Genetic evidence also indicates that both mTOR and AMPK are dispensable for polyposis in PJS,^28,29^ suggesting that other ARK family members contribute to the tumor suppressor functions of LKB1. Salt-inducible kinases (SIKs) are one such family, beyond AMPK, with the potential to mediate the tumor suppressor functions of LKB1—silencing SIKs promotes lung tumor development similar to LKB1 loss,^30–32^ although the mechanism(s) of tumor suppression by SIKs remain to be defined.

More recent work has associated deregulated inflammation as a common feature of LKB1 mutant tumors. PJS mouse models and patient samples exhibit hallmarks of inflammation, including increased infiltration of immune cells (i.e., T cells, macrophages, and neutrophils), elevated levels of inflammatory cytokines (i.e., IL-6 and IL-11), and the activation of signal transducer and activator of transcription 3 (STAT3) in GI polyp tissues.^28,29^ The expression of cyclooxygenase-2, which synthesizes inflammatory prostaglandins, is also elevated in PJS polyps.^33^ Elevated IL-6 and Janus kinase (JAK)-STAT signaling is also a common signature of LKB1- and SIK-deficient NSCLC.^32,34,35^ Evidence suggests that the inflammatory environment fostered by LKB1 mutant tumors promotes immune invasion, in part through recruitment of suppressive myeloid cells (i.e., CD11b^+^Ly6G^+^ neutrophils)^34,35^ and contributes significantly to tumor progression. Inhibiting aberrant inflammation via genetic ablation of *Il6*, dosing animals with IL-6 neutralizing antibodies, or treatment with JAK inhibitors can reduce the growth of LKB1 mutant tumors,^28,29,35^ suggesting that inflammation is a driver—rather than byproduct—of LKB1-dependent tumorigenesis.

As deregulated inflammation is a common feature of tumors promoted by LKB1 mutations, a common etiology linking LKB1 loss and increased inflammatory potential is likely; however, the mechanism(s) by which LKB1 loss promotes deregulated inflammation has remained undefined. Through evaluation of signaling networks downstream of LKB1, we have identified aberrant epigenetic programming—instilled by cAMP response element binding protein (CREB)-regulated transcription coactivator 2 (CRTC2)-CREB signaling downstream of SIKs—as a cell-intrinsic regulator of inflammatory potential, revealing a previously unappreciated anti-inflammatory pathway controlled by LKB1.

## RESULTS

### Loss of LKB1 sensitizes cells to inflammatory stimuli

**Figure 1.**
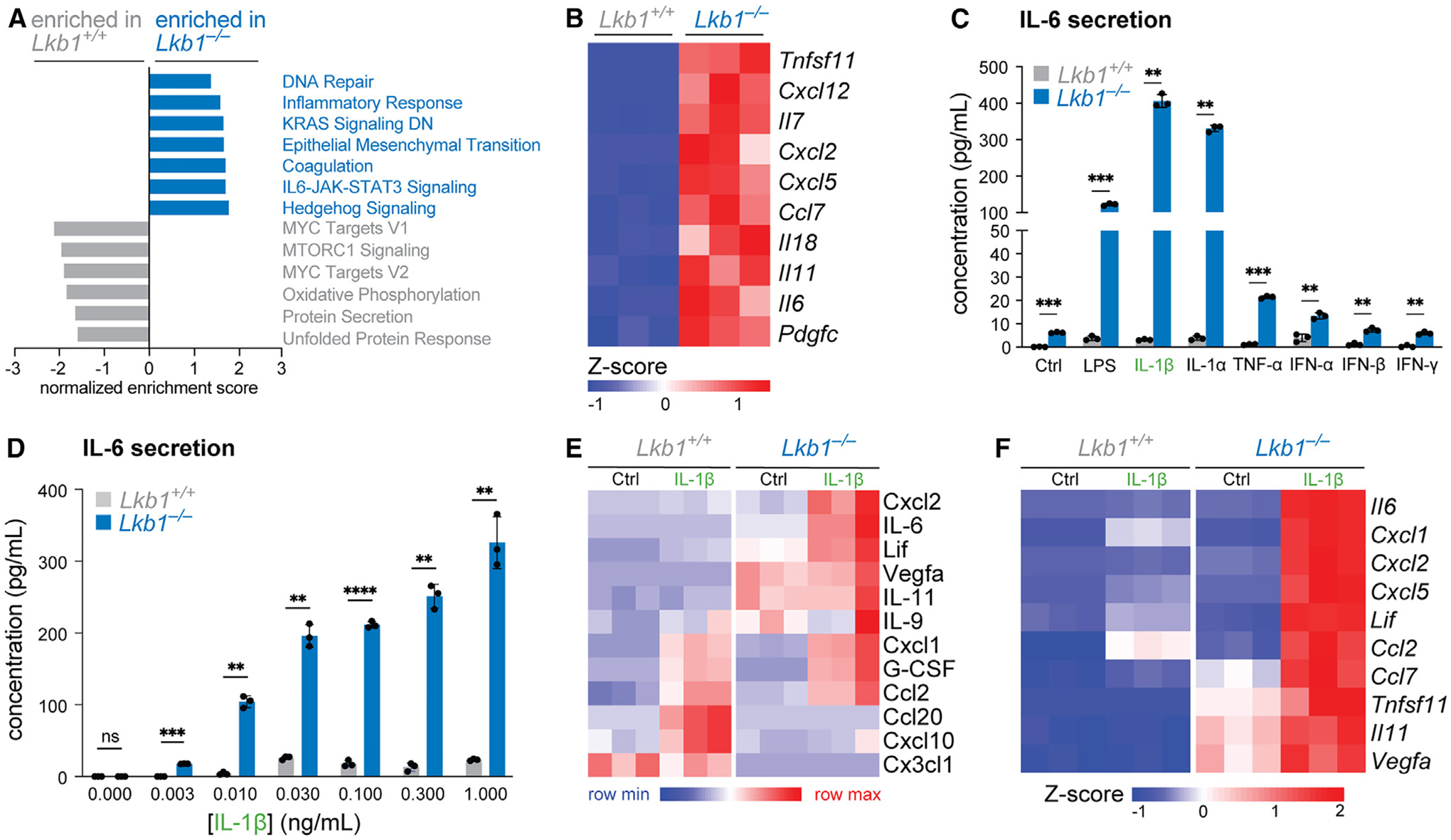
Loss of LKB1 sensitizes cells to inflammatory stimuli (A) Normalized enrichment score for Hallmark pathways enriched in wild-type (*Lkb1*^*+/+*^) and *Lkb1*^−/−^ MEFs. (B) Heatmap of RNA expression (*Z* scores) for the top 10 KEGG cytokine-cytokine receptor interaction genes increased in *Lkb1*^−/−^ MEFs relative to wild type (*Lkb1*^*+/+*^) (n = 3/group). (C) IL-6 production by wild-type (*Lkb1*^*+/+*^) and *Lkb1*^−/−^ MEFs after 6 h stimulation with various inflammatory stimuli (untreated [Ctrl], LPS, IL-1β, IL-1α, TNF-α, IFN-α, IFN-β, or IFN-γ). Data represent the mean ± SD, n = 3/group. (D) IL-6 production by wild-type (*Lkb1*^*+/+*^) and *Lkb1*^−/−^ MEFs stimulated with increasing concentrations of IL-1β for 6 h (mean ± SD, n = 3/group). (E) Heatmap of secreted cytokines/chemokines produced by wild-type (*Lkb1*^*+/+*^) and *Lkb1*^−/−^ MEFs untreated (Ctrl) or stimulated with IL-1β for 6 h (n = 3/group). Shown are secreted cytokines/chemokines with significant differences (p < 0.05) between wild-type and *Lkb1*^−/−^ cells. (F) Heatmap of RNA expression (*Z* scores) for the top 10 IL-1β-induced KEGG cytokine-cytokine receptor genes in wild-type (*Lkb1*^*+/+*^) versus *Lkb1*^−/−^ MEFs (n = 3/group). ns, not significant; **p < 0.01; ***p < 0.001; ****p < 0.0001.

Loss of the tumor suppressor LKB1 is associated with deregulated cell proliferation, cell growth, and survival.^1,36^ However, recent work across diverse tissue types has identified heightened inflammatory signatures as features of LKB1-deficient cells.^28,29,32,35^ To establish a tractable platform to assess the role of LKB1 in control of tissue inflammation, we engineered LKB1-deficient mouse embryonic fibroblasts (MEFs), which respond to diverse inflammatory stimuli by producing acute inflammatory cytokines such as IL-6.^29^ Gene set enrichment analysis (GSEA) of differentially expressed genes between LKB1-deficient MEFs versus their control counterparts (measured by RNA sequencing [RNA-seq]) revealed enrichment of several pathways previously associated with LKB1 loss (i.e., DNA repair, epithelial-mesenchymal transition [EMT], KRAS signaling) and enrichment of genes associated with inflammatory processes, including IL-6-JAK-STAT signaling and cytokine-cytokine receptor interactions (Figures 1A and S1A). Genes encoding prominent members of the IL-6 (i.e., *Il6, Il11, Clcf1*, and *Lif*), chemokine (i.e., *Cxcl12*, *Cxcl2*, *Cxcl5*), and tumor necrosis factor (TNF) (i.e., *Tnfsf11/*RANKL) superfamilies were significantly increased in LKB1-null MEFs at baseline (Figures 1B and S1B). Similarly, PJS polyps also display elevated expression of *Il6* and *Il11*, which are associated with chronic gastric inflammation and GI tumor development,^29,37–39^ as well as *Cxcl2*, a proinflammatory chemokine involved in neutrophil recruitment.^29^ These data corroborate observations of increased inflammatory cytokine expression in LKB1-deficient NSCLC cells,^28,32,35^ validating our model.

To identify the responsiveness of LKB1-deficient cells to inflammatory stimuli, we stimulated *Lkb1*^−/−^ MEFs with diverse inflammatory agonists and measured levels of secreted IL-6 in culture supernatants (Figure 1C). *Lkb1*^−/−^ MEFs produced increased levels of IL-6 at baseline compared with control cells; moreover, *Lkb1*^−/−^ cells displayed heightened IL-6 responses to all agonist classes tested, including lipopolysaccharide (LPS), IL-1 (IL-1α and IL-β), TNF-α, and both type I and II interferons (Figure 1C). Although control MEFs were largely refractory to IL-1α and IL-1β stimulation (Figure 1C), *Lkb1*^−/−^ MEFs were hyper-responsive to IL-1β, producing increasing amounts of IL-6 in a dose-dependent manner (Figure 1D). Using a multiplexed cytokine/chemokine array (and ELISA as confirmation, Figure S1C), we identified leukemia inhibitory factor (LIF) and IL-11 as additional secreted factors produced by *Lkb1*^−/−^, but not wild type, MEFs in response to IL-1β (Figure 1E). In LKB1-deficient cells relative to control, we also observed increased IL-1β-stimulated production of CXCL2, which is expressed in LKB1-mutant PJS polyps, and vascular endothelial growth factor (VEGF), which has previously been shown to be elevated in *Lkb1*^−/−^ fibroblasts^40^ (Figure 1E). Finally, we used RNA-seq to determine whether LKB1 loss influenced the transcription of inflammation-associated genes in response to IL-1β stimulation. Differential expression and GSEA of IL-1β-stimulated genes between *Lkb1*^−/−^ and control MEFs revealed enrichment of cytokine-cytokine receptor genes in LKB1-null cells (Figures S1D and S1E). Expression of IL-6 family cytokines (*Il6, Lif, Il11*), *Tnfsf11/*RANKL, and *Vegfa* was elevated in LKB1-deficient cells at baseline and further elevated upon IL-1β stimulation (Figure 1F).

### LKB1 loss results in hyper-responsiveness to inflammatory stimuli across multiple tissue types

**Figure 2.**
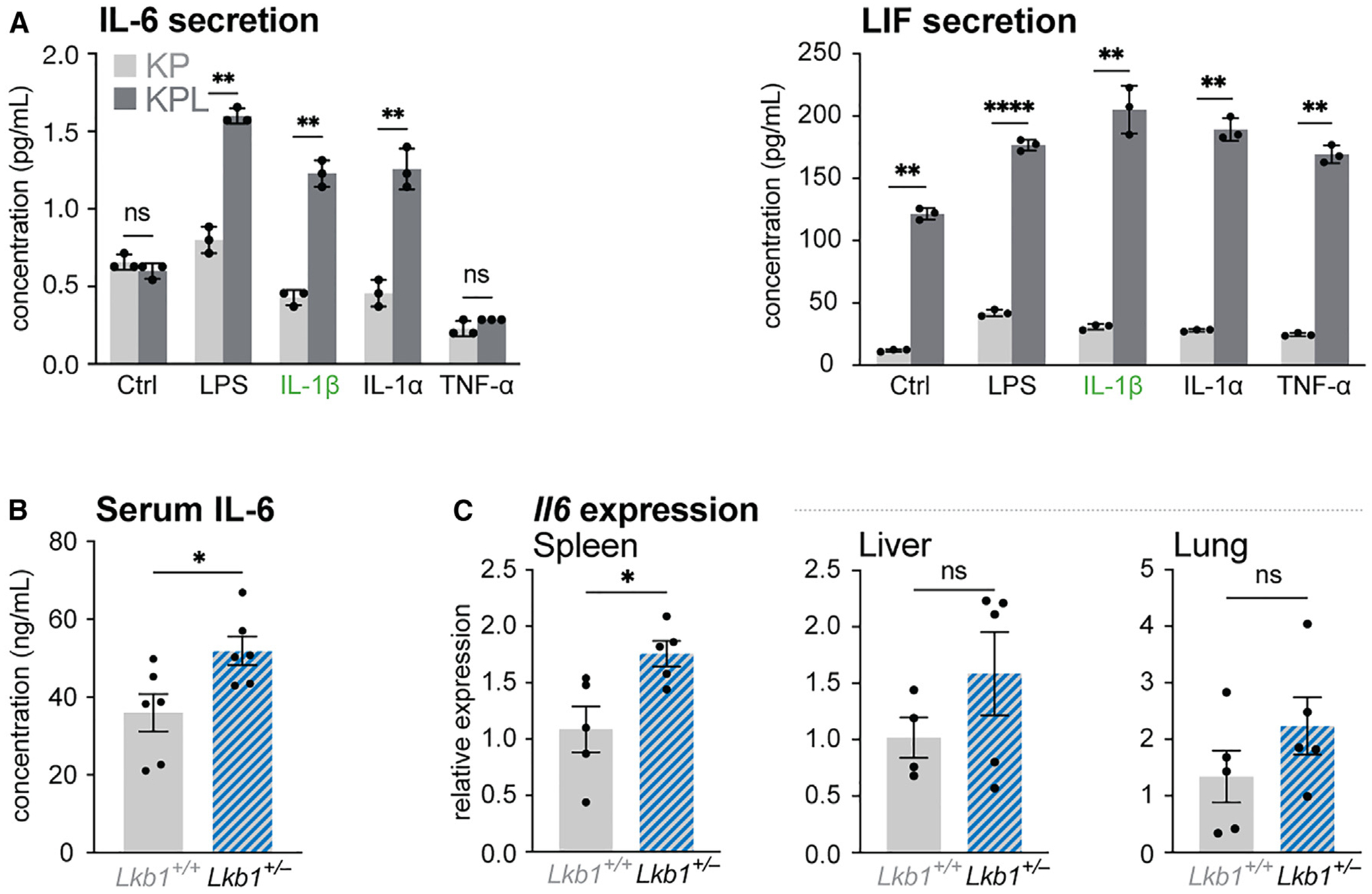
LKB1 loss results in hyper-responsiveness to inflammatory stimuli across multiple tissue types (A) Production of secreted IL-6 (left) and LIF (right) by *Kras*-mutant/*p53*-null (KP) and *Kras*-mutant/*p53*-null/*Lkb1*-null (KPL) NSCLC cells after 6 h stimulation with inflammatory agonists (untreated [Ctrl], LPS, IL-1β, IL-1α, TNF-α). Data represent the mean ± SD, n = 3/group. (B) Serum IL-6 levels from control (*Lkb1*^*+/+*^) and LKB1-mutant (*Lkb1*^*+/−*^) mice 4 h after LPS injection (10 mg/kg). Data represent mean ± SEM for serum samples from 6 individual mice. (C) Normalized relative mRNA levels of *Il6* in the spleen, liver, and lung of control (*Lkb1*^*+/+*^) and LKB1-mutant (*Lkb1*^*+/−*^) mice 4 h after LPS injection (10 mg/kg). Gene expression was made relative to *Tbp* mRNA levels (mean ± SEM, n = 5/group). ns, not significant; *p < 0.05; **p < 0.01; ****p < 0.0001.

We next examined if LKB1 loss in other tissue types also conferred hyper-responsiveness to inflammatory stimuli. LKB1 is commonly mutated in *KRAS*-mutated lung adenocarcinomas,^13^ and previous work identified elevated IL-6-STAT3 signaling in LKB1-deficient NSCLC cells.^32,35^ As such, we stimulated *Kras*-mutant (G12D) NSCLC cells deficient for *p53* (KP) or both *p53* and *Stk11*/LKB1 (KPL) with various inflammatory agonists and found that KPL cells lacking LKB1 displayed increased IL-6 production when exposed to inflammatory agonists, including IL-1β (Figure 2A). Even at baseline, KPL cells displayed ~10-fold higher levels of LIF secretion compared with KP cells, with exposure to inflammatory stimuli further enhancing LIF secretion (Figure 2A). We examined the response of LKB1-mutant tissues to inflammatory stimuli *in vivo* by inducing septic shock in *Lkb1*^*+/−*^ mice via LPS injection. Circulating IL-6 levels were significantly elevated in *Lkb1*^*+/−*^ mice after systemic exposure to LPS, compared with wild-type mice (Figure 2B). Additionally, we observed increased levels of *Il6* mRNA in the spleens of *Lkb1*^*+/−*^ mice relative to *Lkb1*^*+/+*^ mice following LPS exposure (Figure 2C). We observed trends of increased *Il6* mRNA levels in the livers and lungs of *Lkb1*^*+/−*^ mice compared with wild-type mice; however, this did not achieve statistical significance (Figure 2C). Collectively, these data indicate that LKB1 loss results in hyper-responsiveness to a broad range of inflammatory stimuli in different tissue types, suggesting a common mechanism by which LKB1 regulates cell-intrinsic inflammatory potential.

### LKB1 loss sensitizes cells to inflammatory stimuli via deregulated SIK-CRTC2 signaling

**Figure 3.**
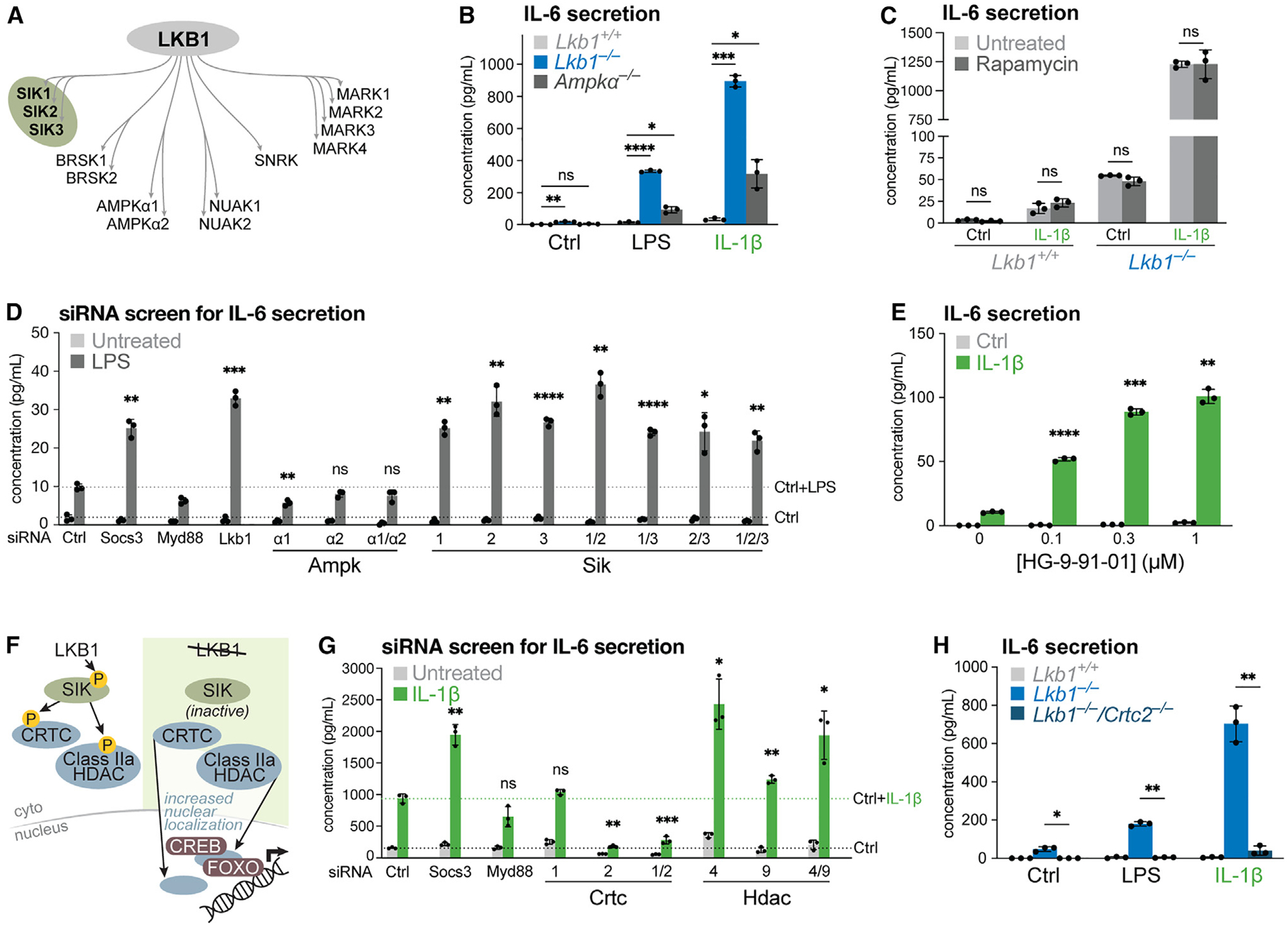
LKB1 loss sensitizes cells to inflammatory stimuli via deregulated SIK-CRTC2 signaling (A) Schematic of AMPK and AMPK-related kinase families regulated by LKB1. (B) IL-6 production by wild-type (*Lkb1*^*+/+*^), *Lkb1*^−/−^, and *Ampkα*^−/−^ MEFs after a 6 h stimulation with the indicated agonists (untreated [Ctrl], LPS, or IL-1β) (mean ± SD, n = 3/group). (C) IL-6 production by wild-type (*Lkb1*^*+/+*^) and *Lkb1*^−/−^ MEFs after a 6 h stimulation with IL-1β. Cells were untreated or treated with rapamycin (50 nM) for 1 h prior to IL-1β treatment (mean ± SD, n = 3/group). (D) IL-6 production by untreated and LPS-stimulated wild-type MEFs transfected with siRNAs targeting *Prkaa1*/*Prkaa2* (Ampkα1 and Ampkα2) and the indicated *Sik* (1/2/3). Non-targeting (Ctrl) and siRNA targeting *Myd88* were used as negative controls. siRNA targeting *Socs3* was used as a positive control. Statistical analysis compared LPS-treated siRNA knockdown to LPS-treated control (Ctrl) (mean ± SD, n = 3/group). (E) IL-6 production by wild-type MEFs after a 6 h stimulation with IL-1β. Cells were treated with a SIK inhibitor (HG-9–91-01) for 1 h prior to IL-1β treatment. Statistical analysis compared IL-1β/SIK inhibitor-treated cells to IL-1β-treated/0 μM HG-9–91-01 (mean ± SD, n = 3/group). (F) Schematic of SIK-CRTC/HDAC signaling downstream of LKB1. CRTCs and HDACs interact with the CREB and Forkhead family (FOXO) transcription factors, respectively. (G) IL-6 production by untreated and IL-1β-stimulated *Lkb1*^−/−^ MEFs transfected with siRNAs targeting the indicated *Crtc* (1/2) or *Hdac* (4/9). Non-targeting (Ctrl) and siRNA targeting *Myd88* were used as negative controls. siRNA targeting *Socs3* was used as a positive control. Statistical analysis compared IL-1β-treated siRNA knockdown to IL-1β-treated control (Ctrl) (mean ± SD, n = 3/group). (H) IL-6 production by wild-type (*Lkb1*^*+/+*^), *Lkb1*^−/−^, and *Lkb1*^−/−^/*Crtc2*^−/−^ MEFs after a 6 h stimulation with LPS, IL-1β, or untreated (Ctrl) (mean ± SD, n = 3/group). ns, not significant; *p < 0.05; **p < 0.01; ***p < 0.001; ****p < 0.0001.

To define the mechanism by which LKB1 loss sensitizes cells to inflammatory stimuli, we investigated downstream signaling. LKB1 is known to regulate the function of 14 downstream kinases, including members of the AMPK and ARK families, which differ in function and/or tissue expression (Figure 3A).^36,41^ MEFs deficient for AMPK activity (via deletion of *Prkaa1* and *Prkaa2* [*Ampkα*^−/−^]) displayed increased LPS- and IL-1β-stimulated IL-6 production compared with controls, but significantly lower levels than observed in LKB1-deficient MEFs (Figure 3B). Loss of LKB1 leads to increased signaling by mTORC1 (Figure S2A). However, IL-1β stimulation did not promote increased activation of the mTORC1 pathway (as measured by phosphorylation of S6-kinase [S6K], ribosomal-S6 protein, or 4E-BP) beyond that induced by serum stimulation (Figure S2B), indicating that mTORC1 signaling is not significantly altered by IL-1β stimulation. Moreover, inhibiting mTORC1 activity with rapamycin did not affect IL-1β-stimulated IL-6 production by *Lkb1*^−/−^ MEFs (Figure 3C). Together, these data suggest that pathways beyond AMPK-mTORC1 signaling regulate inflammatory signaling downstream of LKB1 loss.

To identify LKB1-dependent mediators of IL-6 production, we performed siRNA-mediated screening of AMPK and ARKs in MEFs. SOCS3 is a negative regulator of JAK-STAT signaling that negatively regulates IL-6 signaling *in vivo*.^42,43^ MYD88 is a general adaptor protein that transduces inflammatory signals from Toll-like receptors (TLRs) and IL-1 receptors.^44,45^ Silencing *Socs3* increased, whereas silencing *Myd88* decreased, IL-6 secretion upon LPS stimulation in wild-type MEFs (Figure 3D), validating this assay for identifying potential regulators of IL-6 secretion in response to inflammatory stimuli. Silencing *Prkaa1* (Ampkα1) and/or *Prkaa2* (Ampkα2) did not result in increased baseline or LPS-stimulated IL-6 production, suggesting that acute silencing of AMPK in MEFs does not phenocopy the heightened inflammatory response when LKB1 is silenced in MEFs (Figure 3D). However, silencing members of the SIK (*Sik*1–3) family led to increased LPS-stimulated IL-6 production, similar to that observed when LKB1 is silenced (Figure 3D). To further probe the role of SIK signaling in IL-6 production, we utilized the pan-SIK inhibitor HG-9–91-01, which has an IC_50_ of 0.92, 6.6, and 9.6 nM for SIK1, SIK2, and SIK3, respectively.^46^ Treatment of wild-type MEFs with HG-9–91-01 promoted increased IL-1β-stimulated IL-6 production in a dose-dependent manner (Figure 3E), pointing to LKB1-SIK signaling as a cell-intrinsic regulatory circuit for IL-1β-mediated inflammatory responses.

LKB1 constitutively phosphorylates and activates SIKs, leading to downstream phosphorylation of CREB-regulated transcription coactivators (CRTCs) and class IIa histone deacetylases (HDACs), preventing their translocation to the nucleus (Figure 3F).^47,48^ To determine whether LKB1-dependent inflammatory responses signal through either of these nodes, we performed an siRNA screen targeting HDACs and CRTCs in LKB1-deficient MEFs (Figure 3G). Immunoblotting confirmed that only HDAC4 and HDAC9 are nuclear in *Lkb1*^−/−^ MEFs (Figure S2C). Knockdown of *Crtc2*, alone or in combination with *Crtc1*, reversed the *Lkb1*^−/−^ phenotype of heightened IL-6 production, identifying CRTC2 as the main target downstream of LKB1-SIK signaling promoting inflammatory cytokine expression (Figure 3G). By contrast, the knockdown of *Hdac4* and *Hdac9* enhanced IL-6 production in LKB1-null MEFs, suggesting they may be negative, rather than positive, regulators of IL-6 production in the context of LKB1 deficiency. We next utilized CRISPR-Cas9 editing to generate LKB1/CRTC2 double knockout MEFs (*Lkb1*^−/−^/*Crtc2*^−/−^) (Figure S2D). CRTC2 deletion in *Lkb1*^−/−^ MEFs blocked the production of IL-6 at baseline and in response to LPS and IL-1β treatment (Figure 3H). Overall, these data establish LKB1-SIK-CRTC2 signaling as an anti-inflammatory circuit that regulates IL-6 production in response to diverse inflammatory stimuli.

### LKB1 regulates inflammatory responses via CRTC2-CREB signaling

**Figure 4.**
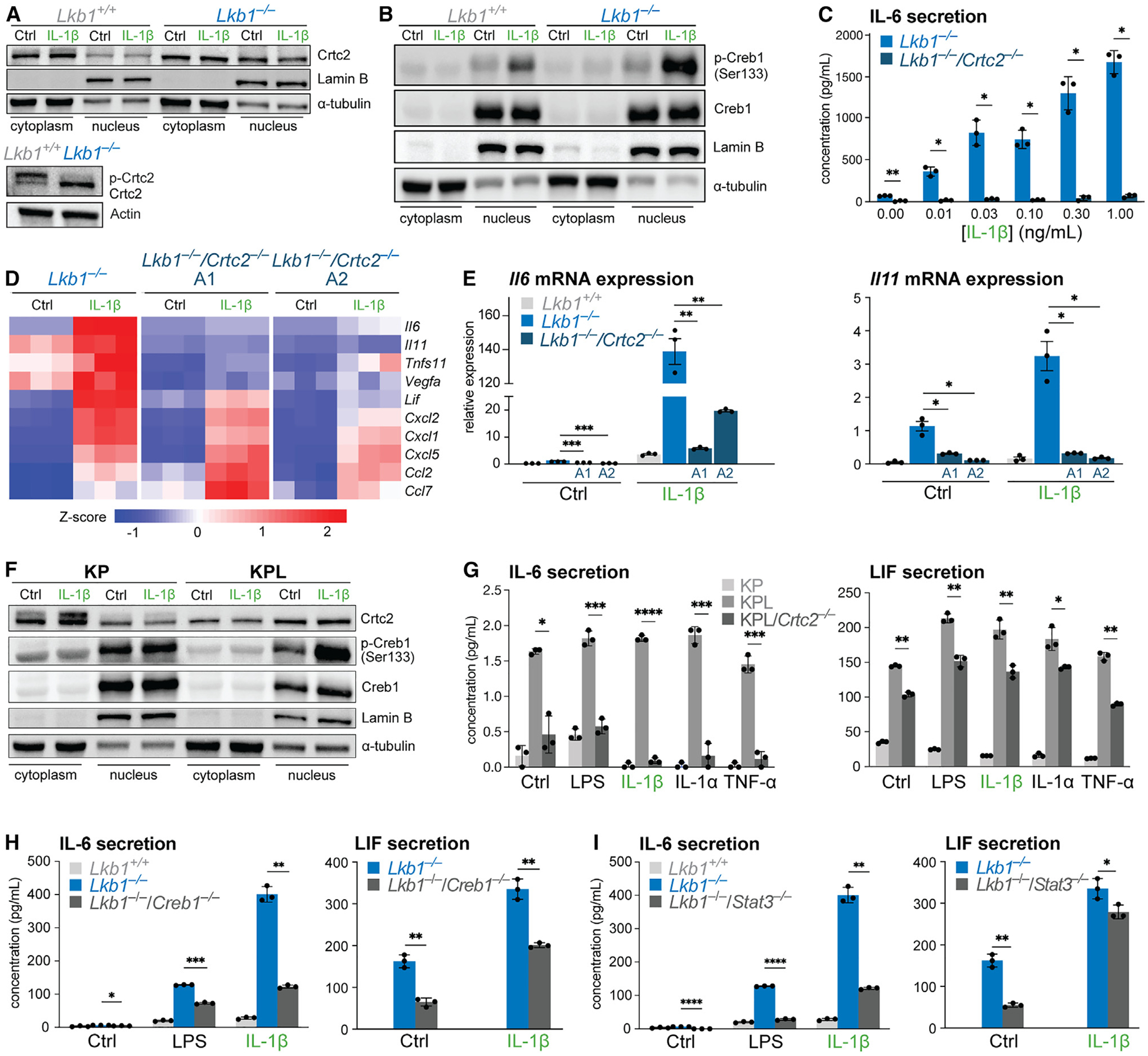
LKB1 regulates inflammatory responses via CRTC2-CREB signaling (A) Top: immunoblot of Crtc2 in cytoplasmic and nuclear fractions from wild-type (*Lkb1*^*+/+*^) and *Lkb1*^−/−^ MEFs stimulated with IL-1β for 30 min. Lamin B and α-tubulin were used as loading controls. Bottom: immunoblot of whole-cell Crtc2 levels in wild-type (*Lkb1*^*+/+*^) and *Lkb1*^−/−^ MEFs. Bands corresponding to phosphorylated (p-Crtc2) and hypo-phosphorylated Crtc2 are indicated. β-actin was used as a loading control. (B) Immunoblot of phosphorylated (Ser133) and total Creb1 in cytoplasmic and nuclear fractions from wild-type (*Lkb1*^*+/+*^) and *Lkb1*^−/−^ MEFs stimulated with IL-1β for 30 min. Lamin B and α-tubulin were used as loading controls. (C) IL-6 production by *Lkb1*^−/−^ and *Lkb1*^−/−^/*Crtc2*^−/−^ MEFs after a 6 h stimulation with increasing doses of IL-1β (mean ± SD, n = 3/group). (D) Heatmap of RNA expression *Z* scores for the top 10 KEGG cytokine-cytokine receptor interaction genes induced by IL-1β in *Lkb1*^−/−^ MEFs relative to *Lkb1*^−/−^/*Crtc2*^−/−^ MEFs. Data from two individual *Lkb1*^−/−^*/Crtc2*^−/−^ cell clones are shown (n = 3/group). (E) Normalized relative mRNA levels of *Il6* and *Il11* in wild-type (*Lkb1*^*+/+*^), *Lkb1*^−/−^, and *Lkb1*^−/−^/*Crtc2*^−/−^ (clone A1/clone A2) MEFs after a 6 h stimulation with IL-1β. Gene expression was made relative to *Tbp* mRNA levels (mean ± SEM, n = 3/group). (F) Immunoblot of Crtc2, phosphorylated (Ser133) Creb1, and total Creb1 in cytoplasmic and nuclear fractions from *Kras*-mutant/*p53*-null (KP) and *Kras*-mutant/*p53*-null/*Lkb1*-null (KPL) NSCLC cells stimulated with IL-1β for 30 min. Lamin B and α-tubulin were used as loading controls. (G) IL-6 (left) and LIF (right) secretion by KP, KPL, and KPL/*Crtc2*^−/−^ NSCLC cells after 6 h stimulation with inflammatory agonists (untreated [Ctrl], LPS, IL-1β, IL-1α, TNF-α) (mean ± SD, n = 3/group). (H) Left: IL-6 production by wild-type (*Lkb1*^*+/+*^), *Lkb1*^−/−^, and *Lkb1*^−/−^/*Creb1*^−/−^ MEFs after a 6 h stimulation with LPS, IL-1β, or untreated (Ctrl). Right: LIF production by *Lkb1*^−/−^ and *Lkb1*^−/−^/*Creb1*^−/−^ MEFs after a 6 h stimulation with IL-1β (mean ± SD, n = 3/group). (I) Left: IL-6 production by wild-type (*Lkb1*^*+/+*^), *Lkb1*^−/−^, and *Lkb1*^−/−^/*Stat3*^−/−^ MEFs after a 6 h stimulation with LPS, IL-1β, or untreated (Ctrl). Right: LIF production by *Lkb1*^−/−^ and *Lkb1*^−/−^/*Stat3*^−/−^ MEFs after a 6 h stimulation with IL-1β (mean ± SD, n = 3/group). *p < 0.05; **p < 0.01; ***p < 0.001; ****p < 0.0001.

We next characterized the mechanism(s) by which CRTC2 regulates inflammatory responses in the context of LKB1 deficiency. When in the nucleus, CRTC2 acts as a coactivator for basic region/leucine zipper motif (bZIP) transcription factors including CREB.^47^ SIK antagonizes CRTC2-CREB signaling by phosphorylating CRTC2 at Ser171, leading to binding by 14–3-3 proteins and sequestration of CRTC2 in the cytoplasm.^49^ Loss of LKB1 inactivates SIK and thus induces CRTC-CREB-dependent transcriptional responses in multiple tissues.^50–52^ Immunoblot analysis of IL-1β-stimulated MEFs revealed that CRTC2 was highly enriched in the nuclear fraction of *Lkb1*^−/−^ MEFs compared with wild-type cells; however, nuclear CRTC2 levels were not affected by IL-1β stimulation (Figure 4A). We also observed hypo-phosphorylation of CRTC2 in LKB1-deficient MEFs (Figure 4A). Examination of CREB1 Ser133 phosphorylation in whole-cell lysates from *Lkb1*^−/−^ cells did not reveal major differences in phospho-CREB1 levels compared with wild-type cells (Figure S3A); however, we observed elevated levels of CREB1 Ser133 phosphorylation specifically in the nuclear fraction of *Lkb1*^−/−^ MEFs following IL-1β treatment compared with wild-type cells (Figure 4B). By contrast, CRTC2 phosphorylation levels were similar between control and AMPK-deficient MEFs, and we observed reduced levels of CREB1 Ser133 phosphorylation (Figure S3A), consistent with previous findings characterizing CREB1 as an AMPK target.^53^ Together, these data are consistent with a model of increased nuclear localization of hypo-phosphorylated CRTC2 in LKB1-deficient cells, which in turn promotes CRTC-CREB-dependent transcriptional responses (Figure 3F). Deleting CRTC2 effectively rendered LKB1-deficient MEFs refractory to inflammatory stimuli, as CRTC2 deletion in *Lkb1*^−/−^ MEFs blocked IL-6 production at all IL-1β concentrations tested (Figure 4C).

To identify LKB1-dependent inflammatory factors regulated by CRTC2, we conducted transcriptional profiling of *Lkb1*^−/−^ MEFs and two different clones of *Lkb1*^−/−^/*Crtc2*^−/−^ MEFs following IL-1β stimulation (Figure 4D). Focusing on genes from the Kyoto Encyclopedia of Genes and Genomes (KEGG) cytokine pathway (from Figure 1), LKB1-regulated inflammatory genes clustered into CRTC2-dependent and CRTC2-independent groups. CRTC2-dependent genes included IL-6 family cytokines (*Il6*, *Il11*, *Lif*), *Vegfa*, and *Tnfsf11*/RankL, while expression of chemokine genes (*Cxcl2*, *Cxcl1*, *Cxcl5*, *Ccl2*) was CRTC2-independent (Figure 4D). Using quantitative PCR (qPCR), we validated that *Il6* and *Il11* expression was reduced at baseline and in response to IL-1β in *Lkb1*^−/−^/*Crtc2*^−/−^ cells compared with *Lkb1*^−/−^ cells (Figure 4E).

We next examined whether CRTC2 mediated the hyper-responsiveness of LKB1-deficient NSCLC cells to inflammatory stimuli. First, we used immunoblots to examine the dynamics of CRTC2 and CREB signaling in KP and KPL NSCLC cells following IL-1β stimulation. Similar to *Lkb1*^−/−^ MEFs, CRTC2 was hypo-phosphorylated (Figure S3B) and highly enriched in the nuclear fraction of KPL cells compared with KP cells (Figure 4F). In response to IL-1β, we observed elevated levels of CREB1 Ser133 phosphorylation in the whole-cell and nuclear fraction of KPL cells compared with KP cells (Figures 4F and S3B). Next, we generated KPL cells that also lack CRTC2 (KPL/*Crtc2*^−/−^) via CRISPR-Cas9 gene editing (validation in Figure S3C). Similar to our results with CRTC2 deletion in MEFs (Figure 4C), deletion of CRTC2 in KPL cells blocked the production of IL-6 and LIF at baseline and in response to several inflammatory agonists (Figure 4G). These findings establish CRTC2 as a common mechanism for the regulation of inflammatory responses downstream of LKB1 across multiple cell types.

The transcription factor CREB1 is known to interact with CRTC2.^54^ To elucidate whether CREB1 works in concert with CRTC2 to regulate inflammatory gene expression downstream of LKB1 loss, we generated LKB1/CREB1 double knockout MEFs (*Lkb1*^−/−^/*Creb1*^−/−^) (validation in Figure S3D). Similar to CRTC2 deletion, CREB1 deletion reduced baseline IL-6 production and blocked LPS- and IL-1β-stimulated IL-6 production in *Lkb1*^−/−^ MEFs (Figure 4H). Production of LIF was also reduced in *Lkb1*^−/−^/*Creb1*^−/−^ cells compared with *Lkb1*^−/−^ cells, both at baseline and in response to IL-1β (Figure 4H). Beyond CREB1, CRTC2 has also been shown to work in tandem with STAT3 to promote gene expression during adipocyte differentiation.^55^ Deleting STAT3 in *Lkb1*^−/−^ MEFs reduced both IL-6 and LIF production compared with *Lkb1*^−/−^ cells with STAT3 signaling intact (Figure 4I). Taken together, these data show that CRTC2 works in concert with CREB1 and STAT3 to mediate the production of inflammatory factors—specifically IL-6 family cytokines—in the context of LKB1 loss.

### LKB1 regulates inflammation-triggered CRTC2-dependent histone acetylation

**Figure 5.**
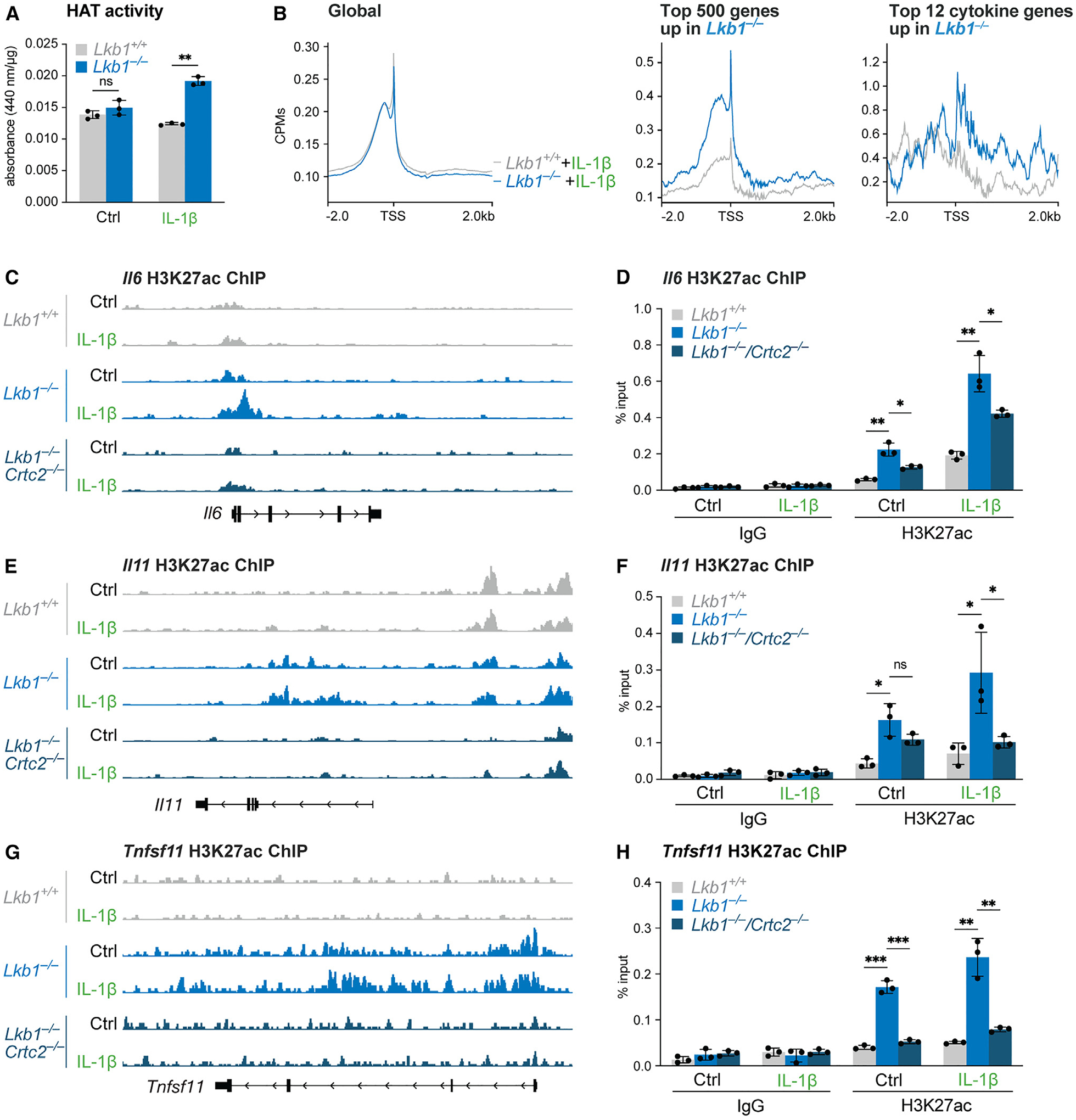
LKB1 regulates inflammation-triggered CRTC2-dependent histone acetylation (A) HAT activity from wild-type (*Lkb1*^*+/+*^) and *Lkb1*^−/−^ nuclear extracts after IL-1β stimulation for 30 min (mean ± SD, n = 3/group). (B) H3K27ac enrichment in relation to transcriptional start sites (TSSs) of genes in the indicated gene sets in wild-type (*Lkb1*^*+/+*^) and *Lkb1*^−/−^ MEFs stimulated with IL-1β for 6 h. (C) Gene tracks for H3K27ac enrichment at the *Il6* locus in wild-type (*Lkb1*^*+/+*^), *Lkb1*^−/−^, and *Lkb1*^−/−^/*Crtc2*^−/−^ MEFs stimulated with IL-1β for 6 h. (D) ChIP-qPCR analysis of H3K27ac enrichment at the *Il6* promoter in wild-type (*Lkb1*^*+/+*^), *Lkb1*^−/−^, and *Lkb1*^−/−^/*Crtc2*^−/−^ MEFs stimulated with IL-1β for 6 h (mean ± SD, n = 3/group). (E) Gene tracks for H3K27ac enrichment at the *Il11* locus in wild-type (*Lkb1*^*+/+*^), *Lkb1*^−/−^, and *Lkb1*^−/−^/*Crtc2*^−/−^ MEFs stimulated with IL-1β for 6 h. (F) ChIP-qPCR analysis of H3K27ac enrichment at the *Il11* promoter in wild-type (*Lkb1*^*+/+*^), *Lkb1*^−/−^, and *Lkb1*^−/−^/*Crtc2*^−/−^ MEFs stimulated with IL-1β for 6 h (mean ± SD, n = 3/group). (G) Gene tracks for H3K27ac enrichment at the *Tnfsf11* locus in wild-type (*Lkb1*^*+/+*^), *Lkb1*^−/−^, and *Lkb1*^−/−^/*Crtc2*^−/−^ MEFs stimulated with IL-1β for 6 h. (H) ChIP-qPCR analysis of H3K27ac enrichment at the *Tnfsf11* promoter in wild-type (*Lkb1*^*+/+*^), *Lkb1*^−/−^, and *Lkb1*^−/−^/*Crtc2*^−/−^ MEFs stimulated with IL-1β for 6 h (mean ± SD, n = 3/group). ns, not significant; *p < 0.05; **p < 0.01; ***p < 0.001.

CRTC2 is part of a multi-subunit transcriptional complex including CREB and the histone acetyltransferases (HATs) CREB-binding protein (CBP) and p300 (CBP/p300).^56^ CBP/p300-dependent acetylation of histone H3 at lysine 27 (H3K27ac) is associated with transcriptional activation, whereas the phosphorylation of CREB at Ser133 promotes increased association with CBP/p300.^57,58^ Given our observation that HDAC4/9 inhibition increased IL-6 production in LKB1-null MEFs (Figure 3G), we hypothesized that CRTC2 may cooperate with CREB and CBP/p300 in LKB1-deficient cells to alter permissive histone acetylation and promote inflammatory gene expression. In an *in vitro* HAT activity assay, we observed a ~40% increase in IL-1β-stimulated HAT activity in *Lkb1*^−/−^ MEFs compared with controls (Figure 5A). Using chromatin immunoprecipitation coupled to next-generation sequencing (ChIP-seq), we observed no difference in global enrichment of H3K27ac at transcriptional start sites (TSSs) between wild-type and *Lkb1*^−/−^ MEFs, either at baseline or following stimulation with IL-1β (Figures 5B and S4A). By contrast, we observed a marked increase in H3K27ac levels around the TSS of the top 500 differentially expressed genes and the top cytokine genes upregulated in IL-1β-stimulated *Lkb1*^−/−^ cells versus the wild type (Figure 5B).

Next, we examined H3K27ac distribution on the inflammatory genes with increased expression in LKB1-deficient cells. On the *Il6* gene (Figure 5C), IL-1β treatment promoted a modest increase in H3K27ac levels around the TSS in wild-type cells. LKB1-deficient MEFs displayed elevated H3K27ac levels at baseline, and IL-1β treatment further increased the acetylation levels (Figure 5C). This trend was verified using ChIP-qPCR, which showed a ~5-fold increase in H3K27ac levels at the *Il6* TSS in *Lkb1*^−/−^ MEFs over the wild type at baseline, and an additional 3-fold increase upon IL-1β stimulation (Figure 5D). Importantly, the ablation of CRTC2 expression in *Lkb1*^−/−^ cells diminished the increased H3K27ac at the *Il6* promoter (Figures 5C and 5D). For the *Il11* gene, whose mRNA expression is stimulated by IL-1β in LKB1-deficient but not wild-type MEFs (Figure 4E), we observed increased H3K27ac levels across the promoter region in *Lkb1*^−/−^ cells at baseline, while the levels were further increased by IL-1β treatment and ablated by CRTC2 deletion (Figures 5E and 5F). By contrast, wild-type cells displayed little H3K27ac enrichment at the *Il11* gene locus, regardless of inflammatory stimulus (Figures 5E and 5F). Finally, we examined H3K27ac enrichment at the *Tnfsf11* locus, as this gene—which encodes RANKL—is elevated in *Lkb1*^−/−^ cells regardless of inflammatory stimulus (Figure 1B). We observed increased H3K27ac levels at the TSS and across the body of the *Tnfsf11* gene in *Lkb1*^−/−^ MEFs at both baseline and IL-1β-stimulated conditions (Figures 5G and 5H). Again, these chromatin features at the *Tnfsf11* locus were absent in wild-type cells and reversed in *Lkb1*^−/−^ cells lacking CRTC2 (Figures 5G and 5H). As a control, we examined H3K27ac levels at the *Gapdh* and *Actb* gene loci, genes whose expression is similar between control, *Lkb1*^−/−^, and *Lkb1*^−/−^/*Crtc2*^−/−^ MEFs, and found similar levels of H3K27ac enrichment at these loci, regardless of genotype or IL-1β treatment (Figure S4B). As such, we conclude that loss of LKB1 promotes increased CRTC2-dependent H3K27ac, altering acetylation-dependent epigenetic programming at inflammatory genes.

### Aberrant histone acetylation drives the inflammatory potential of LKB1-deficient cells

**Figure 6.**
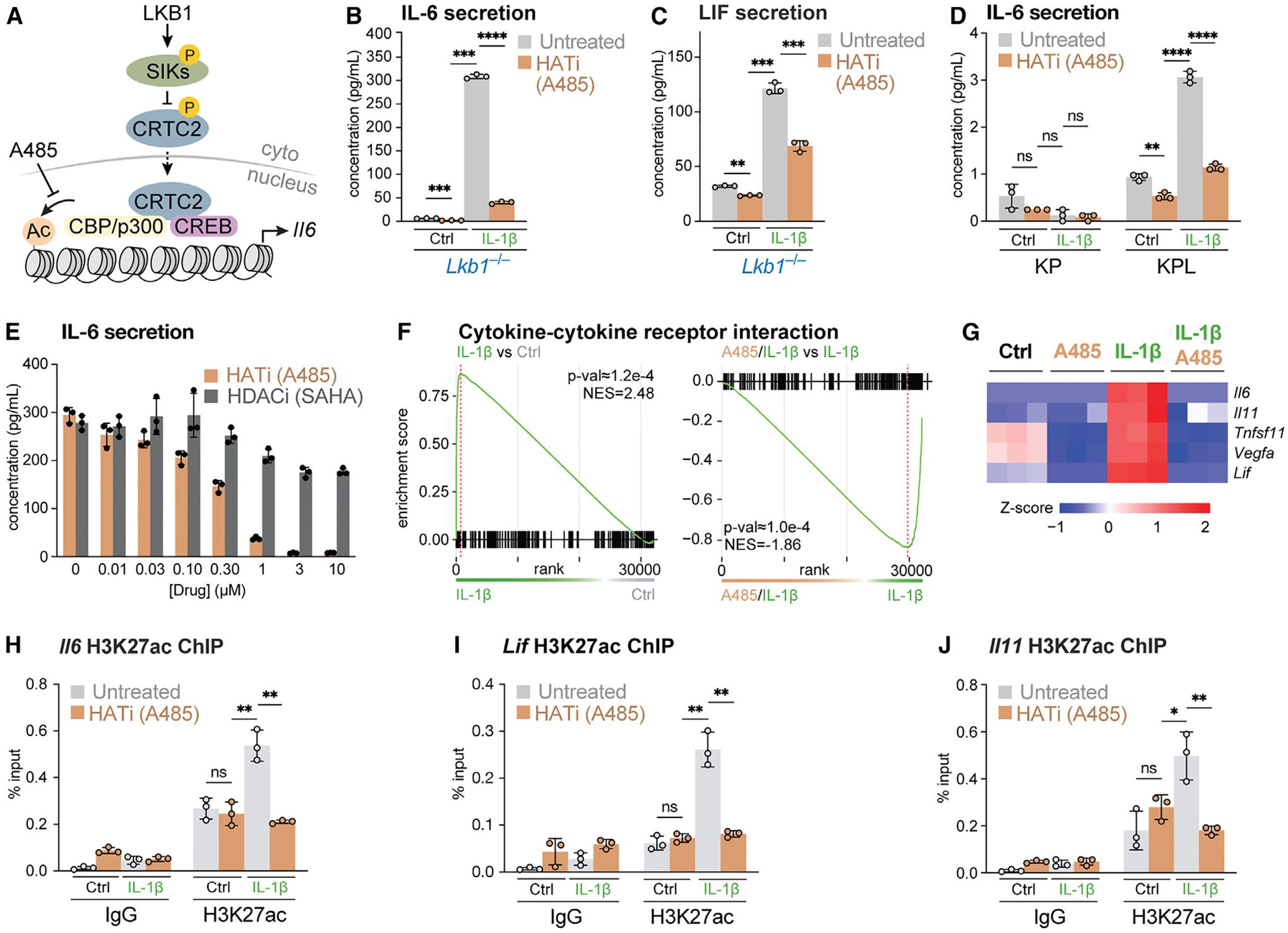
Aberrant histone acetylation drives the inflammatory potential of LKB1-deficient cells (A) Schematic of LKB1 as a negative regulator of histone acetylation via SIK-CRTC2 signaling. (B) IL-6 production by control (Ctrl) or IL-1β-stimulated *Lkb1*^−/−^ MEFs untreated or pre-treated with A485 (1 μM) for 30 min (mean ± SD, n = 3/group). (C) LIF production by control (Ctrl) or IL-1β-stimulated *Lkb1*^−/−^ MEFs untreated or pre-treated with A485 (1 μM) for 30 min (mean ± SD, n = 3/group). (D) IL-6 production by control (Ctrl) or IL-1β-stimulated *Kras*-mutant/*p53*-null (KP) and *Kras*-mutant/*p53*-null/*Lkb1*-null (KPL) NSCLC cells untreated or pre-treated with A485 (1 μM) for 30 min (mean ± SD, n = 3/group). (E) IL-6 production by IL-1β-stimulated *Lkb1*^−/−^ MEFs pre-treated with increasing doses of A485 or vorinostat (SAHA) (mean ± SD, n = 3/group). (F) Gene set enrichment analysis of KEGG cytokine-cytokine receptor interaction genes in control (Ctrl) versus IL-1β-stimulated *Lkb1*^−/−^ MEFs (left) and IL-1β-treated versus IL-1β/A485-treated (1 μM A485) *Lkb1*^−/−^ MEFs (right) (n = 3/group). (G) Heatmap of RNA expression (*Z* scores) for CRTC2-dependent genes in control (Ctrl) or IL-1β-stimulated *Lkb1*^−/−^ MEFs untreated or pre-treated with A485 (1 μM) for 30 min (n = 3/group). (H–J) ChIP-qPCR analysis of H3K27ac enrichment at the (H) *Il6*, (I) *Lif*, and (J) *Il11* promoters in control (Ctrl) or IL-1β-stimulated *Lkb1*^−/−^ MEFs untreated or pre-treated with A485 (1 μM) for 30 min (mean ± SD, n = 3/group). ns, not significant; *p < 0.05; **p < 0.01; ***p < 0.001; ****p < 0.0001.

Increased H3K27ac at inflammatory gene loci in *Lkb1*^−/−^ cells may be attributed to increased histone acetylation or reduced histone deacetylation activities. Downstream of LKB1, SIKs signal to class IIa HDACs in addition to CRTCs (Figure 3F).^48^ To test whether CRTC2 and CBP/p300-dependent histone acetylation drives the increased inflammatory potential of LKB1-deficient cells (Figure 6A), we examined the impact of blocking CBP/p300 HAT activity using the CBP/p300 inhibitor A485.^59–61^ The IC50 of A485 for purified CBP/p300 enzyme is ~0.06 μM, whereas its EC_50_ in cell lines varies between 2 and 6 μM.^59–61^ Treating *Lkb1*^−/−^ MEFs with 1 μM A485 significantly reduced IL-1β-stimulated IL-6 and LIF production (Figures 6B, 6C, and S5A). Similarly, the A485 treatment also reduced IL-1β-stimulated IL-6 production in KPL NSCLC cells (Figure 6D). A485 reduced IL-6 production in *Lkb1*^−/−^ MEFs in a dose-dependent manner, with an EC_50_ of ~400–500 nM and almost complete inhibition at concentrations above 1 μM (Figure 6E). In contrast to blocking CBP/p300, inhibiting HDAC by Vorinostat (aka suberoylanilide hydroxamic acid [SAHA]) did not affect IL-1β-stimulated IL-6 production by *Lkb1*^−/−^ MEFs (Figure 6E).

We next determined the impact of inhibiting CBP/p300 on the transcriptional response of *Lkb1*^−/−^ MEFs following IL-1β stimulation using RNA-seq. Differential gene expression analysis revealed 6,115 genes whose expression was reduced and 5,122 genes whose expression was increased in IL-1β treated *Lkb1*^−/−^ MEFs upon treatment with A485 (Figure S5B). GSEA analysis revealed that the KEGG cytokine-cytokine receptor interaction pathway, which is enriched in IL-1β-stimulated *Lkb1*^−/−^ MEFs compared with wild-type cells, was specifically suppressed in IL-1β-stimulated *Lkb1*^−/−^ MEFs treated with A485 (Figures 6F and S5C). Notably, CRTC2-dependent IL-6 family genes (i.e., *Il6*, *Il11*, *Lif*)—normally elevated in IL-1β-stimulated *Lkb1*^−/−^ MEFs—were all inhibited by A485 treatment (Figure 6G). By contrast, the expression of CRTC2-independent chemokine genes (i.e., *Cxcl1*, *Cxcl2*) was not as drastically impacted by the A485 treatment (Figure S5D).

Mechanistically, we examined the impact of CBP/p300 inhibition on histone acetylation dynamics in LKB1-null cells and found that A485 treatment reversed the IL-1β-stimulated increase in H3K27ac at LKB1-sensitive inflammatory gene loci, including *Il6* (Figure 6H), *Lif* (Figure 6I), and *Il11* (Figure 6J). The reduction in H3K27ac levels correlated with loss of *Il6*, *Lif*, and *Il11* mRNA expression in IL-1β-stimulated *Lkb1*^−/−^ MEFs following A485 treatment (Figure S5E). Together, these data suggest that histone acetylation is a mechanistic driver of increased inflammatory cytokine gene expression in LKB1-deficient cells, an epigenetic mechanism that can be targeted by inhibiting CBP/p300.

## DISCUSSION

Despite the identification of LKB1 as a tumor suppressor over 20 years ago, the etiology of LKB1-dependent tumorigenesis has remained elusive. Although LKB1 loss engenders cells with several critical cancer hallmarks such as apoptosis resistance,^24,31,62,63^ altered metabolism,^64,65^ and therapeutic resistance,^66–68^ recent studies have linked deregulated inflammation as a critical driver of tumor progression in LKB1 mutant tumors.^28,29,35^ Herein, we suggest that CRTC2-dependent epigenetic remodeling of inflammatory gene loci serves as a common mechanism for deregulated inflammation in LKB1 mutant cells. Our data indicate that cell-intrinsic regulation of inflammatory potential is a critical function of LKB1, integrating both metabolic and epigenetic states for inflammation control across multiple cell and tissue types.

Our data reveal four important conclusions about the critical function of LKB1 in inflammation control. First, loss of LKB1 changes the activation threshold of cells toward inflammatory stimuli and does so across multiple cell and tissue types, including epithelial cells and fibroblasts. A common thread is that inflammation-triggered production of the IL-6 family of cytokines (*Il6*, *Il11*, and *Lif*) is highly sensitive to LKB1 status. Importantly, LKB1-deficient cells display increased IL-1β-stimulated production of inflammatory cytokines and chemokines despite normal NF-κB nuclear translocation, suggesting that LKB1 regulates a regulatory circuit—independent of NF-κB—that functions to dampen inflammatory responses.

Second, LKB1 is a critical regulator of inflammatory responses *in vivo*, where LKB1 mutant (*Stk11*^*+/−*^) mice displayed significant increases in circulating IL-6 levels and *Il6* expression in the spleen when challenged with LPS *in vivo*. Similarly, we show that LKB1 mutant lung cancer cells show increased IL-6 production in response to diverse inflammatory stimuli. Increased cytokine secretion is seen in LKB1 mutant tumors *in vivo*, where it is speculated to contribute to immunosuppression and their resistance in the tumor microenvironment.^35^

Third, our data establish deregulated CRTC2-CREB signaling downstream of SIK as the major mechanistic pathway by which LKB1 controls tissue inflammation; silencing either CRTC2 or CREB1 ablates heightened inflammatory responses in LKB1-deficient cells. These observations are consistent with pro-tumorigenic functions of CRTC2 in LKB1 mutant tumors *in vivo*,^32,51^ and may explain how LKB1 loss promotes an immunosuppressive microenvironment in both malignant tumors and PJS polyps.^29,35^

Finally, our data highlight a novel mechanism of LKB1-dependent control of inflammation via deregulated histone acetylation. Aberrant epigenetic remodeling in LKB1 mutant cells, involving both CRTC2 and CBP/p300, functions to increase cell-intrinsic inflammatory potential through enrichment of H3K27ac at inflammatory gene loci. This last observation suggests that LKB1 loss may establish heritable changes in inflammatory potential to daughter cells, which can be further shaped by metabolic conditions and inflammatory cues that impact immune homeostasis in tissues. It also highlights the potential to use epigenetic drugs—such as HAT inhibitors—to target and reverse pathogenic inflammation in tissues driven by LKB1 mutations.

Common features of LKB1-dependent inflammation include deregulation of IL-6 family cytokines^28,29,32,35^ and disruption of type 3 (i.e., T helper [Th]17- versus T regulatory [Treg]-mediated) immune homeostasis.^29,69–71^ IL-6, along with IL-1β and TNF-α, is one of the most prominent proinflammatory cytokines, mediating acute physiologic changes to inflammatory stimuli and chronic inflammation in autoimmunity^72^; yet IL-6 also plays a critical role in wound healing, metabolic homeostasis, and Th17 cell differentiation.^73–75^ LKB1 mutant mice displayed increased inflammatory responses to LPS challenge, marked by heightened IL-6 levels in the serum and elevated *Il6* expression in the spleen (Figure 2). Increased *Il6* transcript levels in the spleen are consistent with the delivery method for LPS via intraperitoneal injection, which is expected to activate innate immune cells in the spleen. These findings indicate a role for LKB1 in the regulation of tissue inflammation. One possible explanation for the lack of increased IL-6 in the lung and liver following systemic exposure to LPS, compared with the exacerbated inflammatory response displayed by *Lkb1*^−/−^ MEFs and KPL cells, is the heterozygous expression of LKB1 in tissues from *Lkb1*^*+/−*^ mice.

Our data indicate that increased CRTC2-CREB signaling downstream of LKB1-SIK drives these increased inflammatory responses (Figure 3F). We speculate that LKB1 normally functions to buffer such cellular responses to inflammatory stimuli by suppressing CRTC2-CREB-mediated transcriptional circuits. Extracellular cues that stimulate protein kinase A (PKA) activity may override this regulatory circuit by both inhibiting SIK function and increasing CREB activity (via Ser133 phosphorylation).^47,76^ Loss of LKB1 would reduce the threshold for inflammatory stimuli to trigger these responses due to unchecked CRTC2 activity. Of note, several of the inflammatory factors produced by IL-1β-stimulated LKB1-null MEFs include cytokines and chemokines found in PJS polyp tissues (i.e., IL-6, IL-11, CXCL2), whereas production of these inflammatory factors is not induced in cells with intact LKB1 signaling (Figures 1E and 1F). IL-6 and IL-11 are associated with chronic gastric inflammation and GI tumor development, and CXCL2 is involved in myeloid cell recruitment.^37–39^ We speculate that chronic tissue inflammation driven by the increased inflammatory potential of LKB1 mutant tissues may serve as a precursor for transformation.

Our data point to CRTC2-dependent regulation of histone acetylation as an essential mediator of LKB1-regulated inflammation. Of note, CRTC2 has previously been implicated in Th17 cell differentiation and CRTC2-deficient mice are protected from experimental autoimmune encephalomyelitis (EAE) induced by pathogenic Th17 cells.^77^ Work using assay for transposase-accessible chromatin coupled to next-generation sequencing (ATAC-seq) has associated SIK with distinct chromatin states in LKB1-deficient primary lung adenocarcinoma,^78^ with the underlying epigenetic modifications influenced by LKB1 undefined. Our studies identify H3K27ac as a defining epigenetic feature of LKB1 mutant cells and establish CRTC2 as the central mediator of this epigenetic process. Enrichment of H3K27ac is not a global feature of LKB1 loss; rather, H3K27ac is enriched specifically at the promoters of genes associated with inflammation in LKB1-null cells (Figure 5B), suggesting that the specificity of histone acetylation is directed by CRTC2-associated signaling complexes.

In our model, CBP/p300 and CRTC2-CREB complexes promote histone acetylation at LKB1-SIK-directed genetic loci, leading to increased H3K27ac levels at inflammatory genes (Figure 6A). Optimal activation of NF-κB target genes (including IL-6) requires CBP/p300,^79^ which may be regulated by CRTC2-CREB complexes. Additionally, CBP/p300 have been shown to promote acetylation of CRTC2 at lysine 628 in the context of fasting, protecting it from degradation.^80^ We speculate that CRTC2 is acetylated by CBP/p300 in LKB1-deficient cells, further promoting its nuclear activity. Since inhibiting the HAT activity of CBP/p300 blocked LKB1-dependent cytokine expression and production, we have identified a druggable target downstream of LKB1 that has the potential to suppress the proinflammatory phenotypes in tissues and cells bearing LKB1 mutations.

Overall, our study reveals a novel role for LKB1 in the epigenetic regulation of inflammatory potential. Our data suggest that LKB1-deficient cells are hyper-responsive to inflammatory stimuli due to deregulated CRTC2 signaling downstream of LKB1 and SIK, leading to abnormal transcription of inflammatory genes. Finally, our results provide a new therapeutic potential for LKB1-associated diseases—including PJS and therapy-resistant LKB1 mutant tumors—by inhibiting the CRTC2-CBP/p300 chromatin-modifying complex.

### Limitations of the study

This work describes a mechanism of LKB1-dependent control of inflammation *in vitro* via deregulated histone acetylation. Further studies are needed to determine the role of CRTC2 and CBP/p300 in LKB1-mediated inflammatory responses *in vivo*, including tumor growth. We also did not assess whether targeting the CRTC2-CREB axis would reduce the production of IL-6 or other inflammatory factors in the microenvironment of LKB1-mutant tumors and whether this would impact the therapeutic resistance of these tumors (i.e., resistance to ICIs). Finally, we did not explore the role of CRTC2 signaling in LKB1 mutant immune cells. These experiments will be the focus of future studies.

## STAR★METHODS

### RESOURCE AVAILABILITY

#### Lead contact

Additional information and request for resources and reagents should be directed to and will be made available by the corresponding author, Russell G. Jones (russell.jones@vai.org).

#### Materials availability

All unique/stable reagents generated in this study will be made available from the lead contact with a completed materials transfer agreement. Plasmids generated in this study will be deposited to Addgene.

#### Data and code availability

- Data tables of differential gene expression analysis and GSEA analysis related to Figures 1 and 4 are shown in Tables S1, S2, S3, and S4. Data tables of differential gene expression analysis and GSEA analysis related to Figure 6 are shown in Tables S5 and S6. RNA-seq data from Figures 1, 4, and 6 and ChIP-seq data from Figure 5 has been deposited at NCBI GEO: GSE214824 and are publicly available as of the date of publication.
- This paper does not report original code.
- Any additional information required to reanalyze the data reported in this paper is available from the lead contact upon request.

### EXPERIMENTAL MODEL AND SUBJECT DETAILS

#### Mice

*Stk11*^+/−^ mice have been previously described.^29^ Mice were bred and maintained under specific pathogen-free conditions at VAI under approved protocols. Experiments were performed using mice between 8 and 12 weeks of age. Both male and female mice were used in this study.

### METHOD DETAILS

#### Cell culture

*Stk11*^−/−^, *Prkaa1;Prkaa2*^−/−^ (Ampkα^−/−^), and wild type 3T3 mouse embryonic fibroblasts (MEFs) have been previously described^29,81^ and tested negative for mycoplasma contamination. MEF cells were cultured in DMEM (Wisent) supplemented with 10% FBS (Wisent), L-glutamine (Gibco), and penicillin–streptomycin (Invitrogen). *Kras*-mutant (G12D) NSCLC cells deficient for *p53* (KP) or both *p53* and *Stk11*/LKB1 (KPL) have been previously described^82^ and tested negative for mycoplasmacontamination. NSCLC cells and were cultured in RPMI 1640 with L-glutamine (Gibco) supplemented with 10% FBS (Wisent) and penicillin–streptomycin (Invitrogen). Cells were treated with LPS *Escherichia coli* K12 (10 μg/mL) (Invivogen, San Diego CA), IL-1β (0.1 ng/mL for MEFs and 10ng/mL for NSCLC cells) (PeproTech), IL-1α (10ng/mL) (R&D Systems), TNF-α (10 ng/mL) (R&D Systems), IFN-α (10 ng/mL) (Biolegend), IFN-β (100 U/mL) (R&D Systems), or IFN-γ (10 ng/mL) (Peprotech) where indicated. Cells were treated with rapamycin (50 nM) (Millipore Sigma), HG-9–91-01 (MedChemExpress), A485 (1μM, unless indicated otherwise) (Tocris Bioscience), and Vorinostat (Selleck Chemicals) where indicated.

#### Cytokine quantification

MEF and NSCLC cell supernatants were collected after the indicated treatments and stored at −80°C. For the multiplex cytokine/chemokine array, supernatants were assayed by the Eve Technologies Mouse Cytokine/Chemokine 44-Plex Discovery Assay Array (Eve Technologies, Calgary, AB). ELISA was used to quantify IL-6 (Invitrogen) and LIF (R&D Systems) production and were performed according to the manufacturer’s instructions.

#### Immunoblotting

For whole cell immunoblotting, cells were lysed in 1X RIPA buffer (Cell Signaling Technology, Danvers, MA) supplemented with protease and phosphatase inhibitors (Roche). Cytoplasmic and nuclear cell fractions were prepared with a nuclear extraction kit according to the manufacturer’s instructions (Abcam). Protein was quantified using a Pierce BCA Protein Assay Kit (Thermo Fisher Scientific, Waltham, MA, USA). Lysates were resolved by SDS-PAGE, transferred to nitrocellulose, and incubated with primary antibodies listed in key resources table and HRP-conjugated secondary antibodies (Cell Signaling Technology).

#### LPS-induced septic shock model

LPS from *E. coli* O111:B4 (Sigma Aldrich) was administered intraperitoneally at a dose of 10 mg/kg. Blood samples were collected, and serum isolated 4 hours post-LPS injection. Serum IL-6 levels were measured via ELISA (Invitrogen) according to the manufacturer’s instructions. Spleen, liver, and lung tissues were harvested 4 hours post-LPS injection and snap frozen in liquid nitrogen. Tissue was ground with a mortar and pestle and total RNA was isolated using a Qiagen RNeasy Mini Kit (QIAGEN, Germantown, MD, USA).

#### siRNA knockdown assays

50 nM Dharmacon ON-TARGETplus SMARTpool siRNA or Non-targeting Control Pool (Horizon Discovery, Cambridge, UK) was incubated with DharmaFECT 1 transfection reagent (Horizon Discovery) in OptiMEM Reduced Serum Media (Gibco) in a 24 well plate at room temperature for 20 min. 5×10^4^ MEFs were seeded onto the transfection mix and incubated at 37°C for 48 hours. After 48 hours, fresh siRNA and transfection reagent mix was incubated in a 6 well plate at room temperature for 20 min. The transfected cells were trypsinized, transferred to the 6 well plate containing new transfection mix, and incubated for another 48 hours. 1.5×10^4^ cells were seeded into a 96 well plate and incubated at 37°C overnight. The next day, cells were stimulated with LPS or IL-1β for 6 hours. Supernatants were collected and IL-6 production was measured via ELISA.

#### CRISPR/Cas9 gene editing

CRISPR sgRNA guide sequences for the genes of interest (*Crtc2*, *Creb1*, *Stat3*) were designed using the Broad Institute GPP sgRNA Design tool. Five sgRNA sequences targeting the first exon were selected for each gene. Oligonucleotide pairs for sgRNAs were synthesized by Integrated DNA Technologies (IDT). Each pair of oligos was annealed and cloned into *Bbs*I-digested pSpCas9(BB)-2A-GFP (PX458) vector (Addgene). Stbl3 competent cells were transformed with the plasmid containing sgRNAs and plated onto agar plates containing ampicillin. Plasmid DNA was isolated from single colonies using a Qiagen HiSpeed Plasmid Maxi kit (Qiagen). MEFs and NSCLC cells were transfected with 2500ng plasmid DNA using Lipofectamine 3000 (Thermo Fisher Scientific) for 48 hours. Fluorescence-activated cell sorting (FACS) was used to isolate GFP-positive cells. Single cell clones were isolated and expanded from the GFP-positive population and gene knockout was validated via western blotting as described.

#### RNA isolation, sequencing, and qPCR analysis

Total RNA was isolated from MEFs using a Qiagen RNeasy Mini Kit (QIAGEN, Germantown, MD, USA). For quantitative PCR (qPCR), total RNA was reverse transcribed using a High Capacity cDNA Reverse Transcriptase kit (Life Technologies) and qPCR performed using SYBR green (Bio-Rad). Libraries for RNA-seq were prepared by the Van Andel Institute (VAI) Genomics Core from 100 ng of total RNA using the KAPA RNA HyperPrep Kit (Kapa Biosystems, Wilmington, MA USA). Ribosomal RNA material was reduced using the QIAseq FastSelect –rRNA HMR Kit (Qiagen, Germantown, MD, USA). RNA was sheared to 300–400 bp. Prior to PCR amplification, cDNA fragments were ligated to Bioo Scientific NEXTflex Adapters (Bioo Scientific, Austin, TX, USA). Quality and quantity of the finished libraries were assessed using a combination of Agilent DNA High Sensitivity chip (Agilent Technologies, Inc.), QuantiFluor^®^ dsDNA System (Promega Corp., Madison, WI, USA), and Kapa Illumina Library Quantification qPCR assays (Kapa Biosystems). Individually indexed libraries were pooled and 50 bp paired-end sequencing was performed on an Illumina NovaSeq6000 sequencer to an average depth of 40M raw paired-reads per transcriptome. Base calling was done by Illumina RTA3 and output of NCS was demultiplexed and converted to FastQ format with Illumina Bcl2fastq v1.9.0. Reads were trimmed to remove low-quality bases and adapter sequences using TrimGalore v0.6.0 (https://github.com/FelixKrueger/TrimGalore) with default settings. Reads were aligned to the GRCm38.p6 reference genome from GENCODE vM24 using STAR v2.7.8a,^83^ which also calculates the per-gene read counts. Read counts were imported into R v4.1.0 and pairwise contrasts were tested using the quasi-likelihood workflow in edgeR v 3.34.0,^84,85^ fitting all the samples together using the model, ‘~0 + group’, where ‘group’ was the combination of the genotype and the treatment. GSEA was performed using clusterProfiler v1.15.1,^86^ ranking genes by −log_10_ of the edgeR-derived P-value multiplied by −1 or 1 based on the log fold change. GSEA plots were generated using enrichPlot v1.15.1 (https://yulab-smu.top/biomedical-knowledge-mining-book/).

#### Chromatin Immunoprecipitation (ChIP) assays

After the indicated treatments, MEFs were crosslinked with 1% formaldehyde for 10 min at room temperature, then quenched with 0.2 M glycine for 5 min at room temperature. Cells were washed twice with cold PBS, collected in PBS with a protease inhibitor cocktail (Sigma Aldrich), then centrifuged at 500g 4°C for 5 min. Cell pellets were snap frozen on dry ice and stored at −80°C. Cells were lysed with lysis buffer (20 mM Tris-HCl, 85 mM KCl, 0.5% NP-40) on ice for 15 minutes and homogenized using a dounce homogenizer. Nuclei were collected by centrifugation at 1,700g 4°C for 5 min and resuspended in shearing buffer (10 mM Tris-HCl, 1mM EDTA, 0.1% SDS). Chromatin was sheared with a Covaris E220 Evolution Focused-Ultrasonicator for 12 min. Chromatin was centrifuged at 10,000g 4°C for 5 min to pellet insoluble material, and supernatant collected. 10 μg of soluble chromatin was incubated with 10μg H3K27ac (Active Motif) or 10μg rabbit IgG (Cell Signaling) at 4°C for 18 hours. Samples were immunoprecipitated with Dynabeads Protein A magnetic beads (Thermo Fisher Scientific) at 4°C for 4 hours and washed. DNA was eluted with elution buffer (100 mM NaHCO_3_, 1% SDS, proteinase K) for 2 hours at 65°C 1000rpm followed by incubation at 95°C for 10 min. DNA was purified with a QIAquick PCR Purification Kit (Qiagen). qPCR was performed using SYBR green (Bio-Rad) and primers for *Il6*, *Il11*, *Lif*, and *Tnfsf11*.

For ChIP sequencing, libraries for Input and IP samples were prepared by the Van Andel Genomics Core from 10 ng of input material and all available IP material using the KAPA Hyper Prep Kit (v5.16) (Kapa Biosystems, Wilmington, MA USA). Prior to PCR amplification, end repaired and A-tailed DNA fragments were ligated to uniquely barcoded dual indexes (IDT, Coralville, IA USA). Quality and quantity of the finished libraries were assessed using a combination of Agilent DNA High Sensitivity chip (Agilent Technologies, Inc.), QuantiFluor^®^ dsDNA System (Promega Corp., Madison, WI, USA), and Kapa Illumina Library Quantification qPCR assays (Kapa Biosystems). 50 bp, paired end sequencing was performed on an Illumina NovaSeq6000 sequencer using an S2, 100 bp sequencing kit (Illumina Inc., San Diego, CA, USA). Base calling was done by Illumina RTA3 and output of NCS was demultiplexed and converted to FastQ format with Illumina Bcl2fastq v1.9.0. Reads were trimmed to remove low-quality bases and adapter sequences using TrimGalore v0.6.0 (https://github.com/FelixKrueger/TrimGalore) with default settings. Reads were aligned to the GRCm38.p6 reference genome from GENCODE vM24 using bwa mem v0.7.17,^87^ marking duplicates using SAMBLASTER v0.1.24.^88^ Alignments were filtered using SAMtools view v1.9^89^ with the parameters ‘-q 30 -F 2828’ and ‘-f 2’. Bigwig files were obtained using the bamCoverage tool in deepTools v3.4.3^90^ with the paramters ‘–binSize 10 –normalizeUsing “CPM” –samFlagExclude 1024 –samFlagInclude 64 –extendReads’, and specifying the ENCODE v2 blacklist^91^ using ‘–blackListFileName’. Coverage profiles and heatmaps were created using the functions, computeMatrix and plotHeatmap, in deepTools v3.4.3. For computeMatrix, the parameters, ‘–missingDataAs-Zero –binSize 10’, were used along with ‘-bl’ to specify the ENCODE v2 blacklist.

#### Histone acetyltransferase activity assay

After IL-1β treatment, nuclear extracts were prepared with a nuclear extraction kit according to the manufacturer’s instructions (Abcam). Nuclear extracts were quantified using a Pierce BCA Protein Assay Kit (Thermo Fisher Scientific). HAT activity was determined using a histone acetyltransferase activity assay kit (Abcam) according to the manufacturer’s instructions. HAT activity is expressed as the relative O.D value per μg of sample.

### QUANTIFICATION AND STATISTICAL ANALYSIS

Data are presented as mean ± SD for technical replicates or mean ± SEM for biological replicates and were analyzed using unpaired Student’s t test (assuming unequal variance). Statistical significance is indicated in all figures by the following annotations: ns, not significant; *, *p* < 0.05; **, *p* < 0.01; ***, *p* < 0.001; ****, p < 0.0001.

## Supplementary Material

Supplemental information

Table S1. Differential gene expression analysis of wild type, LKB1 knockout, and LKB1/CRTC2 knockout MEFs untreated or stimulated with IL-1β, related to Figures 1 and 4

Table S5. Differential gene expression analysis of LKB1 knockout MEFs treated with the HAT inhibitor A485, related to Figure 6

Table S4. Gene set enrichment analysis of Reactome pathways in wild type, LKB1 knockout, and LKB1/CRTC2 knockout MEFs untreated or stimulated with IL-1β, related to Figures 1 and 4

Table S6. Gene set enrichment analysis of KEGG pathways in LKB1 knockout MEFs treated with the HAT inhibitor A485, related to Figure 6

Table S2. Gene set enrichment analysis of Hallmark pathways in wild type, LKB1 knockout, and LKB1/CRTC2 knockout MEFs untreated or stimulated with IL-1β, related to Figures 1 and 4

Table S3. Gene set enrichment analysis of KEGG pathways in wild type, LKB1 knockout, and LKB1/CRTC2 knockout MEFs untreated or stimulated with IL-1β, related to Figures 1 and 4

Supplemental information can be found online at https://doi.org/10.1016/j.molcel.2023.04.017.

## Figures and Tables

**Table:** KEY RESOURCES TABLE

REAGENT or RESOURCE	SOURCE	IDENTIFIER
Antibodies		
Rabbit monoclonal anti-human/mouse beta-actin	Cell Signaling	Cat#4970; RRID: AB_2223172
Rabbit monoclonal anti-Lamin B1	Cell Signaling	Cat#12586; RRID: AB_2650517
Rabbit polyclonal anti-alpha-tubulin	Cell Signaling	Cat#2144; RRID: AB_2210548
Rabbit monoclonal anti-LKB1	Cell Signaling	Cat#3047; RRID: AB_2198327
Rabbit polyclonal anti-CRTC2	Proteintech	Cat#12497-1-AP; RRID: AB_2260910
Rabbit polyclonal anti-CREB1	Proteintech	Cat#12208-1-AP; RRID: AB_2245417
Rabbit monoclonal anti-phospho-CREB (Ser133)	Cell Signaling	Cat#9198; RRID: AB_2561044
Rabbit monoclonal anti-phospho-p70 S6 kinase (Thr389)	Cell Signaling	Cat#97596; RRID: AB_2800283
Rabbit monoclonal anti-p70 S6 kinase	Cell Signaling	Cat#2708; RRID: AB_390722
Rabbit polyclonal anti-phospho-S6 ribosomal protein (Ser235/236)	Cell Signaling	Cat#2211; RRID: AB_331679
Rabbit monoclonal anti-S6 ribosomal protein	Cell Signaling	Cat#2217; RRID: AB_331355
Rabbit monoclonal anti-phospho-4EBP1 (Thr37/46)	Cell Signaling	Cat#2855; RRID: AB_560835
Rabbit monoclonal anti-phospho-HDAC4 (Ser246)/HDAC5 (Ser259)/HDAC7 (Ser155)	Cell Signaling	Cat#3443; RRID: AB_2118723
Rabbit polyclonal anti-phospho-HDAC4 (Ser246)/HDAC5 (Ser259)/HDAC9 (Ser220)	Invitrogen	Cat#PA5-37835; RRID: AB_2554443
Rabbit monoclonal anti-HDAC4	Cell Signaling	Cat#15164; RRID: AB_2798733
Rabbit monoclonal anti-HDAC5	Cell Signaling	Cat#20458; RRID: AB_2713973
Rabbit monoclonal anti-HDAC7	Cell Signaling	Cat#33418; RRID: AB_2716756
Rabbit polyclonal anti-HDAC9	Invitrogen	Cat#PA5-90264; RRID: AB_2805993
Goat anti-rabbit IgG, HRP-linked	Cell Signaling	Cat#7074; RRID: AB_2099233
Histone H3K27ac	Active Motif	Cat#39133; RRID: AB_2561016
Normal Rabbit IgG	Cell Signaling	Cat#2729; RRID: AB_1031062
Bacterial and Virus Strains		
One Shot Stbl3 Chemically Competent *E. coli*	Invitrogen	Cat#C737303
Chemicals, Peptides, and Recombinant Proteins		
DMEM	Wisent Bioproducts	Cat#319-015-CL
RPMI Medium 1640	Gibco	Cat#11875-093
Opti-MEM I	Gibco	Cat#31985070
HBSS	Gibco	Cat#14185-052
Nu-Serum IV Culture Supplement	Corning	Cat#355504
Penicillin-streptomycin	Gibco	Cat#15070-063
L-Glutamine	Gibco	Cat#25030-081
LPS from *E. coli* 0111:B4	Sigma-Aldrich	Cat#L2630
Standard LPS, *E. coli* K12	Invivogen	tlrl-eklps
Recombinant murine IL-1β	Peprotech	Cat#211-11B
Recombinant mouse IL-1α	R&D Systems	Cat#400-ML
Recombinant mouse TNF-α	R&D Systems	Cat#410-MT
Recombinant mouse IFN-α	BioLegend	Cat#752802
Recombinant mouse IFN-β	R&D Systems	Cat#12410-1
Recombinant murine IFN-γ	Peprotech	Cat#315-05
Rapamycin	Millipore Sigma	Cat#37094
A 485	Tocris Bioscience	Cat#6387
Vorinostat	Selleck Chemicals	Cat#S1047
HG-9-91-01	MedChemExpress	Cat#HY-15776
BbsI	NEB	Cat#R0539
Dynabeads Protein A	Invitrogen	Cat#10001D
Proteinase K	Invitrogen	Cat#25530-015
Pierce 16% Formaldehyde (w/v), methanol-free	Thermo Fisher	Cat#28908
Critical Commercial Assays		
Qiagen RNAeasy Mini Kit	Qiagen	Cat#74104
QIAquick PCR Purification Kit	Qiagen	#28104
HiSpeed Plasmid Maxi Kit	Qiagen	#12662
Histone acetyltransferase activity assay kit	Abcam	#ab65352
Mouse IL-6 ELISA kit	Thermo Fisher	#88-7064-88
Mouse LIF Quantikine ELISA kit	R&D Systems	MLF00
Mouse Cytokine/Chemokine 44-Plex Discovery Assay Array	Eve Technologies	MD44
cOmplete, Mini, EDTA-free Protease Inhibitor Cocktail	Roche	11836170001
PhosSTOP	Roche	4906845001
Nuclear extraction kit	Abcam	ab113474
ON-TARGETplus Mouse Stk11 siRNA	Horizon Discovery	L-044342-00-0005
ON-TARGETplus Mouse Socs3 siRNA	Horizon Discovery	L-040626-01-0005
ON-TARGETplus Mouse Myd88 siRNA	Horizon Discovery	L-063057-00-0005
ON-TARGETplus Mouse Prkaa1 siRNA	Horizon Discovery	L-041035-00-0005
ON-TARGETplus Mouse Prkaa2 siRNA	Horizon Discovery	L-040809-00-0005
ON-TARGETplus Mouse Sik1 siRNA	Horizon Discovery	L-044399-00-0005
ON-TARGETplus Mouse Sik2 siRNA	Horizon Discovery	L-041008-00-0005
ON-TARGETplus Mouse Sik3 siRNA	Horizon Discovery	L-061908-00-0005
ON-TARGETplus Mouse Crtc1 siRNA	Horizon Discovery	L-066810-01-0005
ON-TARGETplus Mouse Crtc2 siRNA	Horizon Discovery	L-049303-00-0005
ON-TARGETplus Mouse Hdac4 siRNA	Horizon Discovery	L-043626-00-0005
ON-TARGETplus Mouse Hdac9 siRNA	Horizon Discovery	L-066143-00-0005
ON-TARGETplus Non-targeting Control Pool	Horizon Discovery	D-001810-10-05
DharmaFECT 1 Transfection Reagent	Horizon Discovery	Cat#T-2001
Deposited data		
RNA-sequencing and ChIP-sequencing data	This manuscript	GEO: GSE214824
Experimental Models: Cell Lines		
Wild type mouse embryonic fibroblasts (MEFs)	Faubert et al.^64^	N/A
Lkb1^−/−^ MEFs	Faubert et al.^64^	N/A
*Ampk*^−/−^ MEFs	Rabinovitch et al.^81^	N/A
*Lkb1*/Crtc2^−/−^ MEFs	This manuscript	N/A
*Lkb1*/Creb1^−/−^ MEFs	This manuscript	N/A
*Lkb1*/Stat3^−/−^ MEFs	This manuscript	N/A
WT/Crtc2^−/−^ MEFs	This manuscript	N/A
*Kras*-mutant/*p53*-null (KP) NSCLC cells	Liu et al.^82^	N/A
*Kras*-mutant/*p53*-null/*Lkb1*-null (KPL) NSCLC cells	Liu et al.^82^	N/A
*Kras*-mutant/*p53*-null/*Lkb1*-null/*Crtc2*-null (KPL/Crtc2^−/−^) NSCLC cells	This manuscript	N/A
Experimental Models: Organisms/Strains		
Stk11^+/−^ mice	Poffenberger et al.^29^	N/A
Recombinant DNA		
pSpCas9(BB)-2A-GFP (PX458)	Addgene	Addgene #48138
Software and Algorithms		
GraphPad Prism V6 or V7	GraphPad Software	www.graphpad.com

## Highlights

- Loss of LKB1 sensitizes cells to diverse inflammatory stimuli
- LKB1 regulates inflammatory responses via CRTC2-CREB signaling
- Loss of LKB1 promotes increased CRTC2-dependent H3K27ac at inflammatory gene loci
- Increased histone acetylation boosts inflammatory potential in LKB1-deficient cells
