# Supplementary material for: LKB1 controls inflammatory potential through CRTC2-dependent histone acetylation
Source: Mol Cell. Author manuscript; Available in PMC 2026 May 1. (PMC13133714; doi:10.1016/j.molcel.2023.04.017)
Supplement: Supplemental information [file NIHMS2160715-supplement-Supplemental_information.pdf]

**Molecular Cell, Volume *83***

## Supplemental information

# LKB1 controls inflammatory potential through CRTC2-dependent histone acetylation

**Shelby E. Compton, Susan M. Kitchen-Goosen, Lisa M. DeCamp, Kin H. Lau, Batsirai Mabvakure, Matthew Vos, Kelsey S. Williams, Kwok-Kin Wong, Xiaobing Shi, Scott B. Rothbart, Connie M. Krawczyk, and Russell G. Jones**

**Inventory of Supplemental Information**

**Supplemental Tables**

**Table S7**, related to Figure 3 and Figure 4. List of CRISPR sgRNA guides used to generate *Crtc2*, *Creb1*, and *Stat3* knockout cells.

**Table S8**, related to Figure 4 and Figure 6. List of qPCR primers used for mRNA expression analysis.

**Table S9**, related to Figure 5 and Figure 6. List of ChIP-qPCR primers used in this study.

**Supplemental Figures**

**Figure S1**, Related to Figure 1. Loss of LKB1 sensitizes cells to inflammatory stimuli.

**Figure S2**, Related to Figure 3. LKB1 loss sensitizes cells to inflammatory stimuli via deregulated SIK-CRTC2 signaling.

**Figure S3**, Related to Figure 4. LKB1 regulates inflammatory responses via CRTC2-CREB signaling.

**Figure S4**, Related to Figure 5. LKB1 regulates inflammation-triggered CRTC2-dependent histone acetylation.

**Figure S5,** Related to Figure 6. Aberrant histone acetylation drives the inflammatory potential of LKB1-deficient cells.

**Table S7**, **related to Figure 3 and Figure 4. List of CRISPR sgRNA guide sequences used to generate *Crtc2*, *Creb1*, and *Stat3* knockout cells.**

| Gene | Forward | Reverse |
|---|---|---|
| *Crtc2* | CACCGTGATGGACATCGGCTCCACA | AAACTGTGGAGCCGATGTCCATCAC |
| *Creb1* | CACCGCAGCTGCACTAAGGTTACAG | AAACCTGTAACCTTAGTGCAGCTGC |
| *Stat3* | CACCGCAAAGAGTCACATGCCACGT | AAACACGTGGCATGTGACTCTTTGC |

**Table S8, related to Figure 4 and Figure 6. List of qPCR primers used for mRNA expression analysis.**

| Gene | Forward | Reverse |
|---|---|---|
| *Il6* | GAGGATACCACTCCCAACAGACC | AAGTGCATCATCGTTGTTCATACA |
| *Il11* | GGGGCCTGCTGTTGTTAAAG | TCCTGGAACTCACGAACTCAC |
| *Lif* | GTCTTGGCCGCAGGGATTG | GCACAGGTGGCATTTACAGG |

**Table S9, related to Figure 5 and Figure 6. List of ChIP-qPCR primers used in this study.**

| Gene | Forward | Reverse |
|---|---|---|
| *Il6* | CACTTCACAAGTCGGAGGCT | AATGAATGGACGCCCACACT |
| *Il11* | TGGTCCCCTCTAGCTATGCC | AAAGAGAGGTGCGGACAGAG |
| *Lif* | CTCAGGCCACCAACTTCAGA | TTCAGGGTGACACCATCGTT |
| *Tnfsf11* | TGAATACGACCAACTGCGCT | GGAGGAAGCTCCGTGCTAAA |

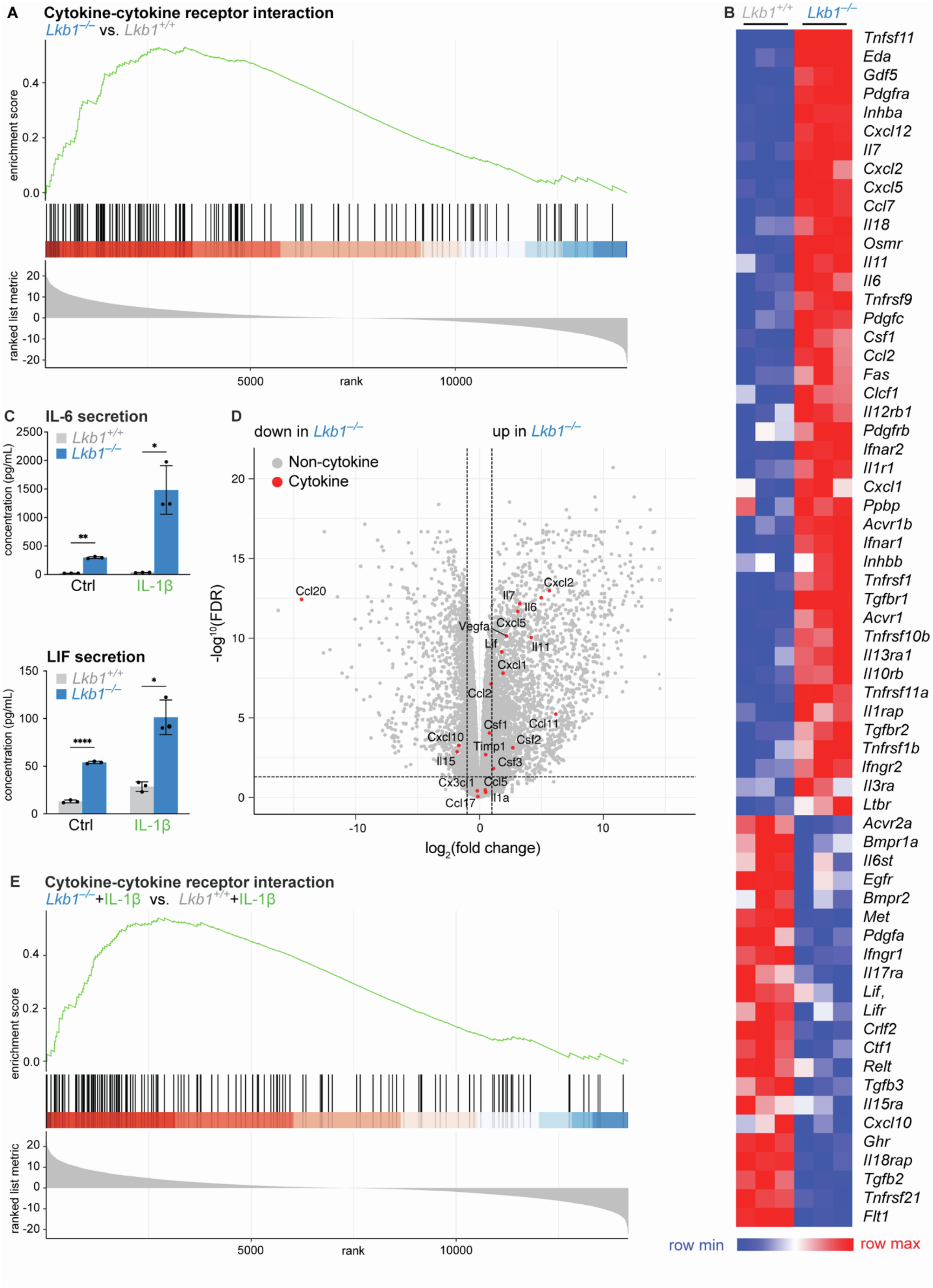

A
Cytokine-cytokine receptor interaction
Lkb1−/− vs. Lkb1+/+
enrichment score
0.4
0.2
0.0
ranked list metric
20
10
0
-10
-20
5000
rank
10000
B
Lkb1+/+
Lkb1−/−
Tnfsf11
Eda
Gdf5
Pdgfra
Inhba
Cxcl12
Il7
Cxcl2
Cxcl5
Ccl7
Il18
Osmr
Il11
Il6
Tnfrsf9
Pdgfc
Csf1
Ccl2
Fas
Clcf1
Il12rb1
Pdgfrb
Ifnar2
Il1r1
Cxcl1
Ppbp
Acvr1b
Ifnar1
Inhbb
Tnfrsf1
Tgfbr1
Acvr1
Tnfrsf10b
Il13ra1
Il10rb
Tnfrsf11a
Il1rap
Tgfbr2
Tnfrsf1b
Ifngr2
Il3ra
Ltbr
Acvr2a
Bmpr1a
Il6st
Egfr
Bmpr2
Met
Pdgfa
Ifngr1
Il17ra
Lif,
Lifr
Crlf2
Ctf1
Relt
Tgfb3
Il15ra
Cxcl10
Ghr
Il18rap
Tgfb2
Tnfrsf21
Flt1
row min
row max
C
IL-6 secretion
Lkb1+/+
Lkb1−/−
concentration (pg/mL)
2500
2000
1500
1000
500
0
**
*
Ctrl
IL-1β
LIF secretion
Lkb1+/+
Lkb1−/−
concentration (pg/mL)
150
100
50
0
****
*
Ctrl
IL-1β
D
down in Lkb1−/−
up in Lkb1−/−
Non-cytokine
Cytokine
-log10(FDR)
20
15
10
5
0
Ccl20
Cxcl2
Il7
Il6
Vegfa
Cxcl5
Lif
Il11
Cxcl1
Ccl2
Ccl11
Csf1
Cxcl10
Csf2
Timp1
Il15
Csf3
Cx3cl1
Ccl5
Ccl17
Il1a
-10
0
10
log2(fold change)
E
Cytokine-cytokine receptor interaction
Lkb1−/−+IL-1β vs. Lkb1+/++IL-1β
enrichment score
0.4
0.2
0.0
ranked list metric
20
10
0
-10
-20
5000
rank
10000

**Figure S1**, **Related to Figure 1. Loss of LKB1 sensitizes cells to inflammatory stimuli.**

**A,** Gene set enrichment analysis (GSEA) of cytokine-cytokine receptor interaction genes in untreated wild type (*Lkb1*$^{+/+}$) versus *Lkb1*$^{-/-}$ MEFs (n=3/group). **B,** Heatmap of differentially expressed cytokine-cytokine receptor interaction genes in untreated wild type (*Lkb1*$^{+/+}$) versus *Lkb1*$^{-/-}$ MEFs (n=3/group). **C,** IL-6 (*top*) and LIF (*bottom*) production by wild type (*Lkb1*$^{+/+}$) and *Lkb1*$^{-/-}$ MEFs after a 6-hour stimulation with IL-1β (mean±SD, n=3/group). **D,** Volcano plot showing cytokine and chemokine gene expression ($log_2$(fold change)) versus statistical significance (-log10(FDR)) in *Lkb1*$^{-/-}$ versus wild type MEFs stimulated with IL-1β. Cytokine/chemokine genes are highlighted by red dots (n=3/group). **E,** GSEA plot of cytokine-cytokine receptor interaction genes in wild type (*Lkb1*$^{+/+}$) versus *Lkb1*$^{-/-}$ MEFs stimulated with IL-1β for 6h (n=3/group).

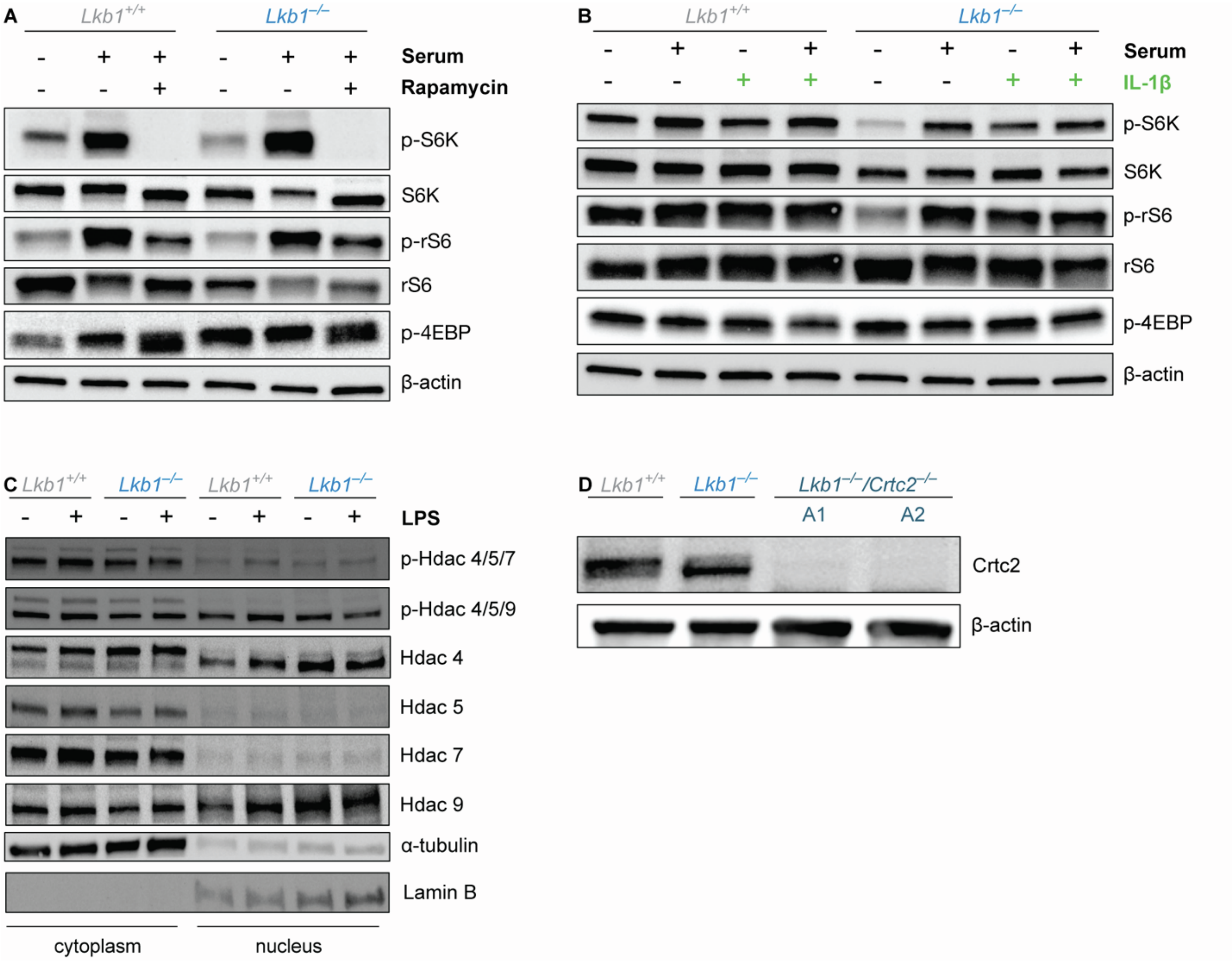


**Figure S2, Related to Figure 3. LKB1 loss sensitizes cells to inflammatory stimuli via deregulated SIK-CRTC2 signaling.**

**A,** Immunoblots of mTOR signaling components in wild type (*Lkb1*$^{+/+}$) and *Lkb1*$^{-/-}$ MEFs. Cells were serum starved (0.5% FBS) for 24 h and had 10% FCS serum addback (+) or no serum addback (-) for 1 h prior to harvesting cell lysates. Cells were treated with rapamycin (50 nM, added at the time of serum addback) as a control for mTORC1 activity. **B,** Immunoblots of mTORC1 signaling components in wild type (*Lkb1*$^{+/+}$) and *Lkb1*$^{-/-}$ MEFs stimulated with IL-1β after 24 h serum starvation. Cells were serum starved for 24 h, followed by serum addback with or without IL-1β stimulation for 1 h. **C,** Immunoblots for phospho-Hdac4/5/7, phospho-Hdac4/5/9, and total Hdac 4, 5, 7, 9 in

nuclear versus cytoplasmic fractions in wild type (*Lkb1*$^{+/+}$) and *Lkb1*$^{-/-}$ MEFs after 30 min of LPS stimulation. **D,** Immunoblot for Crtc2 in wild type (*Lkb1*$^{+/+}$), *Lkb1*$^{-/-}$, and *Lkb1*$^{-/-}$/*Crtc2*$^{-/-}$ MEFs.

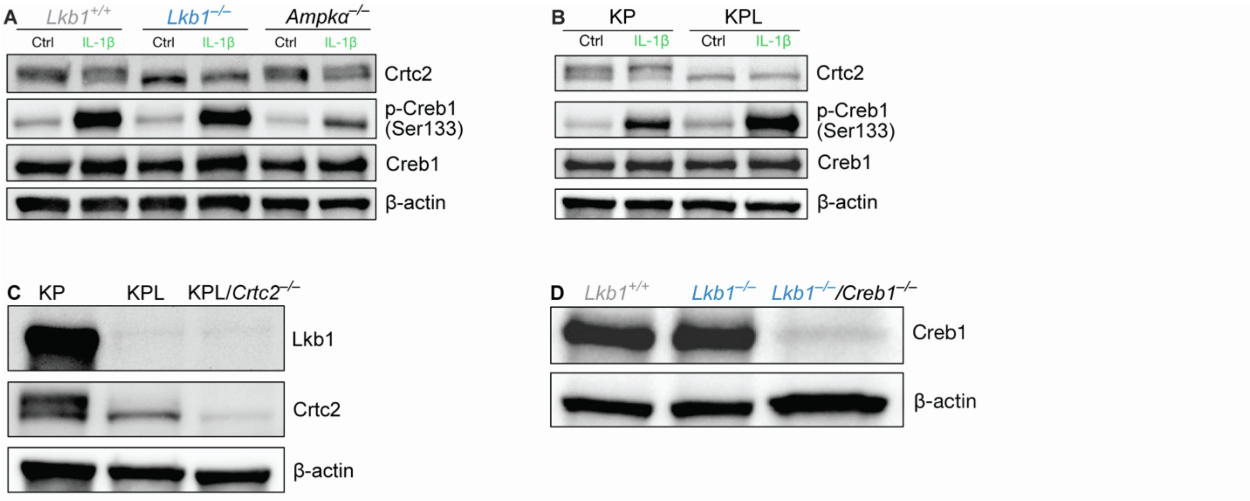


**Figure S3, Related to Figure 4. LKB1 regulates inflammatory responses via CRTC2-CREB signaling.**

**A,** Immunoblot of whole-cell Crtc2, phosphorylated (Ser133) Creb1, and total Creb1 in wild type (*Lkb1*$^{+/+}$), *Lkb1*$^{-/-}$, and *Ampk*$\alpha^{-/-}$ MEFs stimulated with IL-1β for 30 min. **B,** Immunoblot of whole-cell Crtc2, phosphorylated (Ser133) Creb1, and total Creb1 in *Kras*-mutant/*p53*-null (KP) and *Kras*-mutant/*p53*-null/*Lkb1*-null (KPL) NSCLC cells stimulated with IL-1β for 30 min. **C,** Immunoblot for Lkb1 and Crtc2 in KP, KPL, and KPL/*Crtc2*$^{-/-}$ NSCLC cells. **D,** Immunoblot for Creb1 in wild type (*Lkb1*$^{+/+}$), *Lkb1*$^{-/-}$, and *Lkb1*$^{-/-}$/*Creb1*$^{-/-}$ MEFs. β-actin levels are shown as loading controls for protein levels in lysates.

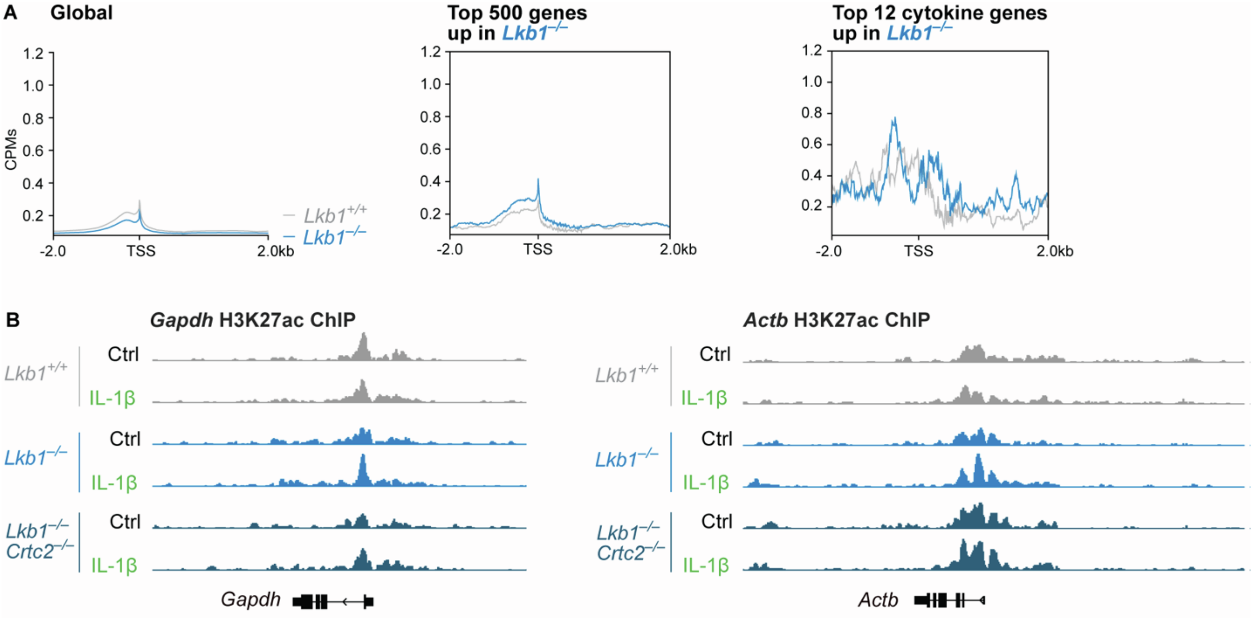


**Figure S4, Related to Figure 5. LKB1 regulates inflammation-triggered CRTC2-dependent histone acetylation.**

**A,** H3K27ac enrichment in relation to transcription start sites (TSS) of genes in the indicated gene sets in unstimulated wild type (*Lkb1*$^{+/+}$) and *Lkb1*$^{-/-}$ MEFs. H3K27ac enrichment is depicted as the counts per million (CPM) across the TSS and gene body (±2 kb). **B,** Gene tracks for H3K27ac enrichment at the *Gapdh* and *Actb* loci in wild type (*Lkb1*$^{+/+}$), *Lkb1*$^{-/-}$, and *Lkb1*$^{-/-}$/*Crtc2*$^{-/-}$ MEFs stimulated without (Ctrl) or with IL-1β for 6 hours.

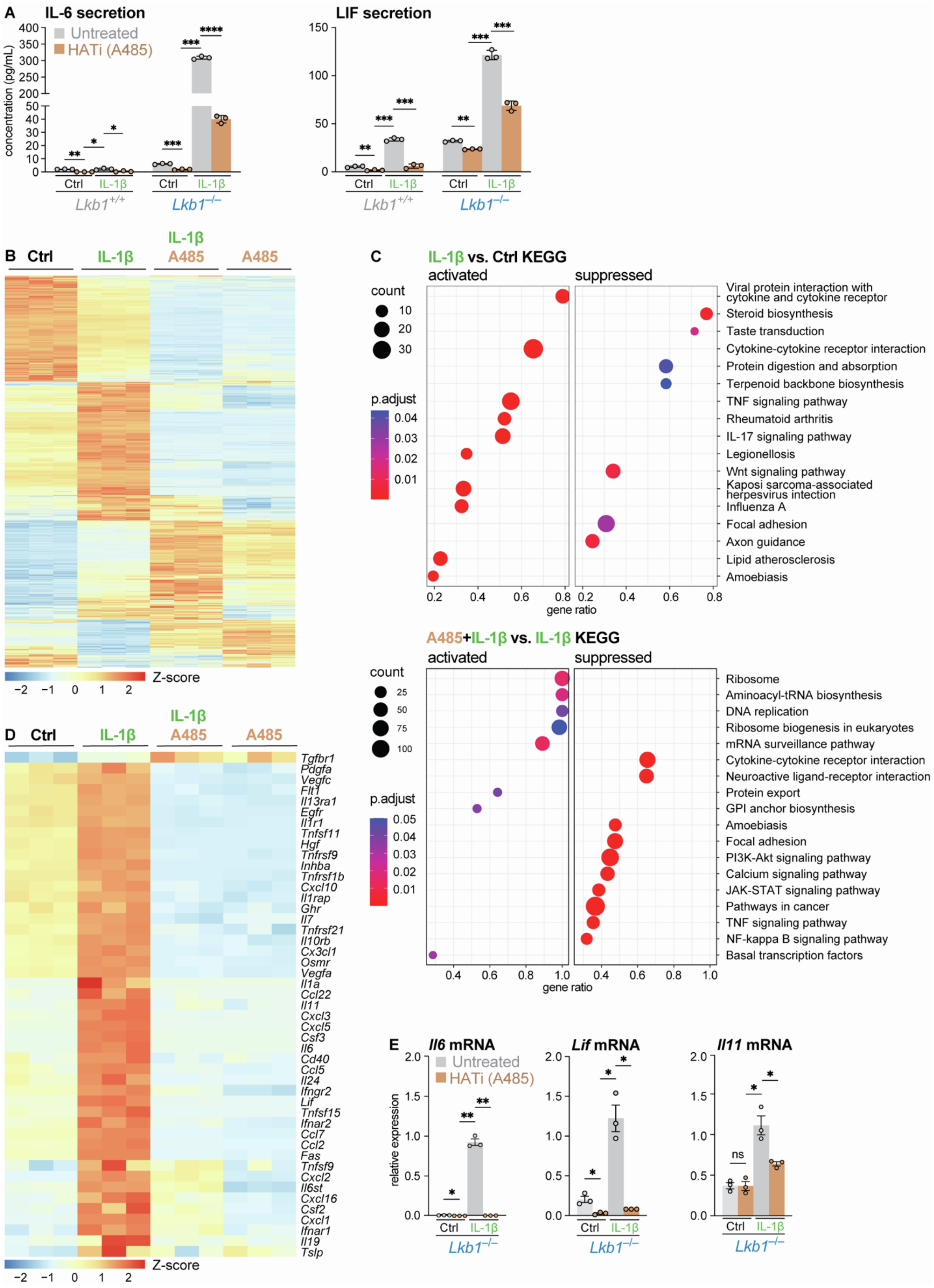
A
IL-6 secretion
Untreated
HATi (A485)
concentration (pg/mL)
Ctrl
IL-1β
Lkb1+/+
Lkb1−/−
LIF secretion
B
Ctrl
IL-1β
IL-1β A485
A485
Z-score
C
IL-1β vs. Ctrl KEGG
activated
suppressed
count
p.adjust
gene ratio
Viral protein interaction with cytokine and cytokine receptor
Steroid biosynthesis
Taste transduction
Cytokine-cytokine receptor interaction
Protein digestion and absorption
Terpenoid backbone biosynthesis
TNF signaling pathway
Rheumatoid arthritis
IL-17 signaling pathway
Legionellosis
Wnt signaling pathway
Kaposi sarcoma-associated herpesvirus infection
Influenza A
Focal adhesion
Axon guidance
Lipid atherosclerosis
Amoebiasis
A485+IL-1β vs. IL-1β KEGG
Ribosome
Aminoacyl-tRNA biosynthesis
DNA replication
Ribosome biogenesis in eukaryotes
mRNA surveillance pathway
Cytokine-cytokine receptor interaction
Neuroactive ligand-receptor interaction
Protein export
GPI anchor biosynthesis
Amoebiasis
Focal adhesion
PI3K-Akt signaling pathway
Calcium signaling pathway
JAK-STAT signaling pathway
Pathways in cancer
TNF signaling pathway
NF-kappa B signaling pathway
Basal transcription factors
D
Tgfbr1
Pdgfa
Vegfc
Flt1
Il13ra1
Egfr
Il1r1
Tnfsf11
Hgf
Tnfrsf9
Inhba
Tnfrsf1b
Cxcl10
Il1rap
Ghr
Il7
Tnfrsf21
Il10rb
Cx3cl1
Osmr
Vegfa
Il1a
Ccl22
Il11
Cxcl3
Cxcl5
Csf3
Il6
Cd40
Ccl5
Il24
Ifngr2
Lif
Tnfsf15
Ifnar2
Ccl7
Ccl2
Fas
Tnfsf9
Cxcl2
Il6st
Cxcl16
Csf2
Cxcl1
Ifnar1
Il19
Tslp
E
Il6 mRNA
Lif mRNA
Il11 mRNA
relative expression
ns

**Figure S5, Related to Figure 6. Aberrant histone acetylation drives the inflammatory potential of LKB1-deficient cells.**

**A,** IL-6 (*left*) and LIF (*right*) production by control (Ctrl) or IL-1β-stimulated wild type (*Lkb1*$^{+/+}$) and *Lkb1*$^{-/-}$ MEFs untreated or pre-treated with A485 (1μM) for 30 min (mean±SD, n=3/group). **B,** Heatmap of RNA levels (z-score) for all significant (p-value <0.05) differentially expressed genes between control versus IL-1β-stimulated *Lkb1*$^{-/-}$ MEFs. Z-scores are shown for control (Ctrl), IL-1β-treated, IL-1β/A485-treated, and A485-treated cells (n=3/group). **C,** Gene set enrichment analysis (GSEA) dot plots for KEGG pathways activated or suppressed in IL-1β-treated versus control (Ctrl) (*top*) and IL-1β/A485-treated (1μM A485) versus Il-1β-treated (*bottom*) *Lkb1*$^{-/-}$ MEFs. **D,** Heatmap of RNA levels (z-scores) for genes in the KEGG cytokine-cytokine receptor interaction pathway that are significantly (p-value <0.05) differentially expressed between control and IL-1β-treated *Lkb1*$^{-/-}$ MEFs. Z-scores are shown for control (Ctrl), IL-1β-treated, IL-1β/A485-treated, and A485-treated cells (n=3/group). **E,** Relative mRNA levels of *Il6*, *Lif*, and *Il11* in control (Ctrl) or IL-1β-stimulated *Lkb1*$^{-/-}$ MEFs untreated or pre-treated with A485 (1μM) for 30 min. Gene expression was made relative to *Tbp* mRNA levels in each sample (mean±SEM, n=3/group).
